# Fission Yeast CSL Transcription Factors: Mapping Their Target Genes and Biological Roles

**DOI:** 10.1371/journal.pone.0137820

**Published:** 2015-09-14

**Authors:** Martin Převorovský, Martina Oravcová, Jarmila Tvarůžková, Róbert Zach, Petr Folk, František Půta, Jürg Bähler

**Affiliations:** 1 Research Department of Genetics, Evolution & Environment and UCL Cancer Institute, University College London, London, United Kingdom; 2 Department of Cell Biology, Faculty of Science, Charles University in Prague, Prague, Czech Republic; Newcastle University, UNITED KINGDOM

## Abstract

**Background:**

Cbf11 and Cbf12, the fission yeast CSL transcription factors, have been implicated in the regulation of cell-cycle progression, but no specific roles have been described and their target genes have been only partially mapped.

**Methodology/Principal Findings:**

Using a combination of transcriptome profiling under various conditions and genome-wide analysis of CSL-DNA interactions, we identify genes regulated directly and indirectly by CSL proteins in fission yeast. We show that the expression of stress-response genes and genes that are expressed periodically during the cell cycle is deregulated upon genetic manipulation of *cbf11* and/or *cbf12*. Accordingly, the coordination of mitosis and cytokinesis is perturbed in cells with genetically manipulated CSL protein levels, together with other specific defects in cell-cycle progression. Cbf11 activity is nutrient-dependent and *Δcbf11*-associated defects are mitigated by inactivation of the protein kinase A (Pka1) and stress-activated MAP kinase (Sty1^p38^) pathways. Furthermore, Cbf11 directly regulates a set of lipid metabolism genes and *Δcbf11* cells feature a stark decrease in the number of storage lipid droplets.

**Conclusions/Significance:**

Our results provide a framework for a more detailed understanding of the role of CSL proteins in the regulation of cell-cycle progression in fission yeast.

## Introduction

Fission yeast, *Schizosaccharomyces pombe*, has been instrumental in the identification of fundamental principles of cell-cycle control [1]. The cell cycle is regulated at multiple levels by numerous protein factors that integrate proliferative and anti-proliferative signals, both intra- and extra-cellular. Key post-translational regulators are the Cdc2^CDK1^ cyclin-dependent kinase, and the Wee1 kinase and Cdc25 phosphatase, which regulate Cdc2 activity and mitotic entry in a negative and positive manner, respectively. At the transcriptional level, several transcription factors have been identified that regulate specific clusters of periodically expressed genes during the cell cycle [2–4]. These factors include the forkhead proteins Sep1 and Fkh2, and the MADS box protein Mbx1 (active at M phase; role in mitosis and cytokinesis), the zinc-finger protein Ace2 (M/G1 phase; role in cell separation), the MBF complex and its negative regulators Nrm1 and Yox1 (G1/S phase; role in DNA replication), and the GATA-type transcription factor Ams2 (S phase; expression of histone genes) [5–13]. Combinatorial regulation by multiple transcription factors has been suggested for many periodically expressed genes [4], and, importantly, for a substantial number of cell cycle-regulated genes the regulator(s) controlling their expression remain unknown. Potential novel transcriptional regulators have been suggested by computational network analysis of gene expression data [14]. Our understanding of the mechanisms driving periodic gene expression during the cell cycle is far from complete.

CSL proteins comprise a family of transcription factors that regulate development and cell fate decisions in Metazoa, mostly via the Notch signalling pathway [15]. Depending on context, Notch/CSL also promote or inhibit cell-cycle progression by regulating the expression of several cyclins and CDK inhibitors [16–19]. CSL family members are also present in many fungi, but their functions are much less understood [20,21]. We previously identified two antagonistic CSL paralogs in *S*. *pombe*, Cbf11 and Cbf12, that function as transcription activators and recognize the canonical metazoan CSL response element on DNA [21–23]. Fission yeast CSL proteins have been implicated in the coordination of cell and nuclear division, cell morphology, and regulation of cell adhesion [22,24]. Mutants with altered *cbf11* or *cbf12* gene dosage have recently been identified in screens for altered cell size and shape, phenotypes associated with aberrant cell-cycle progression [25,26]. Furthermore, the stability of *cbf12* mRNA is negatively regulated by the tristetraprolin RNA-binding protein Zfs1 [27], which plays a role in cell adhesion, cell size determination, and the coordination of mitosis and cytokinesis [28,29]. Collectively, these data raise the intriguing possibility of an important role for Cbf11 and Cbf12 in the regulation of cell-cycle progression in fission yeast.

Previously, Chua and colleagues reported expression microarray data for *cbf11* deletion and *cbf12* overexpression under a single growth condition (rich and minimal medium, respectively), and ChIP-chip data for ectopically overexpressed Cbf12. The authors used the data to identify CSL target genes relevant to cell flocculation and described the regulatory roles of CSL proteins therein [24]. Here we set out to systematically identify CSL target genes under a range of growth conditions. We show that Cbf11 and Cbf12 contribute directly and indirectly to the regulation of distinct sets of genes, including stress-response genes and genes expressed periodically during the cell cycle, and we show that Cbf11 directly regulates a group of lipid metabolism genes. We further show that the function of Cbf11 in regulating cell-cycle progression is affected by nutrients and by protein kinase A (Pka1) and stress-activated MAP kinase (Sty1) pathways.

## Materials and Methods

### Yeast culture and transformation

Fission yeast cells were grown according to standard procedures [30] at 30 or 32°C, unless stated otherwise, in either rich yeast extract with supplements (YES) or Edinburgh minimal medium (EMM; Formedium). A list of fission yeast strains used in this study is provided in S1 Table. The lithium acetate method was used for transformation [31]. Overexpression of *cbf11* and *cbf12* from a plasmid was regulated by the presence (repression) or absence (induction) of 15 μM thiamine in EMM [32]. Routine optical density (OD) measurements of liquid cell cultures were taken using the WPA CO 8000 Cell Density Meter (Biochrom). Growth curves were measured in the VarioSkan Flash instrument (Thermo Scientific) using 12-well dishes and 1.4 ml culture volumes. To assay growth on solid media, exponentially growing cells were 10-fold serially diluted and spotted onto YES plates.

### Microscopy

For cell size and septation index measurements, exponentially growing cells were fixed in 10% formaldehyde (in PBS) for 15 min, washed three times with PBS, stained with DAPI (1 μg/ml) and/or calcofluor (50 μg/ml) and subjected to fluorescence microscopy using a Zeiss Axiophot microscope and the OpenLab software (PerkinElmer), or the Olympus CellR system. Cell length at division was determined using the ImageJ 1.45 software [33].

For quantification of the occurrence of catastrophic mitosis, exponentially growing cells were fixed in 70% ethanol, rehydrated in water, stained with DAPI and photographed using the Olympus CellR system.

The procedure for neutral lipid droplet quantification was adapted from [34]. Live cells growing exponentially in YES were stained with Nile red (10 μM; Sigma) for 10 min at room temperature and images were taken using the Olympus CellR system (GFP filter; excitation 475 nm, emission 530 nm). Lipid droplets were counted manually for at least 200 cells per sample and the number of droplets was normalized to cell volume using the ImageJ software (cylindrical approximation of cell shape was used for calculation) [33].

### Plasmids and constructs

The lists of oligonucleotides and plasmids used in this study are provided in S2 Table and S3 Table, respectively. Plasmids for inducible overproduction of Cbf11 and Cbf12 under the control of the full-strength *nmt1* promoter were constructed as follows. The respective CSL cDNAs were re-cloned from the previously described plasmids pJR08 (*cbf11*) and pMP32 (*cbf12*) [22], using the Expand High Fidelity PCR system (Roche) and primer pairs mp59 & mp60 and mp61 & mp62, respectively. The resulting PCR products were cloned into the pGEM T-Easy vector (Promega), verified by sequencing, and inserted into the PstI/NotI sites of the pJR2-3XU vector [32].

The fission yeast knock-in strain expressing C-terminally triple HA-tagged Cbf11 from its endogenous chromosomal locus (MP26) was constructed in a wild-type background (JB32) by standard PCR-mediated one-step gene tagging using the pFA6a-3HA-natMX6 plasmid as template [35]. The mp41 forward primer consisted of 80 nt complementary to the 3' end of the *cbf11* ORF (stop codon not included) and 20 nt complementary to the 5' end of the 3HA-natMX6 tagging cassette. The reverse mp55 primer contained 80 nt complementary to the genomic sequence starting 80 nt downstream of the *cbf11* ORF (the 80 nt gap was introduced to obtain a primer with a higher melting temperature) and 20 nt complementary to the 3' end of the 3HA-natMX6 tagging cassette.

The strain expressing C-terminally double TAP-tagged Cbf11 from its endogenous chromosomal locus (MP15) was constructed analogously in an auxotrophic background (PN559). The mp41 forward primer and a reverse primer complementary to the region immediately downstream (i.e., without the 80 nt gap as for the mp55 primer) of the *cbf11* ORF (mp42) were used for the amplification of the tagging cassette from the pFA6-CTAP4-natMX6 plasmid [35].

To obtain a *Δcbf11*::*natR* strain, the natMX6 cassette conferring nourseothricin (clonNAT) resistance was amplified from the pFA6-13Myc-NatMX6 plasmid template [35] using primers mp57 and mp58 (these universal primers are complementary to promoter and terminator regions, respectively, of various derivatives of the MX6 cassette and may be used for other combinations of marker switching). The resulting PCR product was integrated by homologous recombination into an auxotrophic *Δcbf11*::*kanR* strain (MP23), and the resulting nourseothricin-resistant G418-sensitive strain (MP34) was verified by PCR.

Deletion of *cbf11* in sterile strains was carried out using the pMP91 targeting plasmid based on pCloneNAT1 as described [36]. All other strains used in this study were constructed by standard genetic crosses.

### Gene expression analysis by microarrays

For transcriptome profiling of CSL knock-outs, either cells growing exponentially in EMM or YES media (4 × 10^6^ cells/ml), or cells reaching early stationary phase (4.5 × 10^7^ cells/ml) were used. For CSL overexpression analysis, strains harbouring the required plasmids were grown in EMM with thiamine to late exponential phase, washed twice in EMM without thiamine, diluted and grown further in EMM without thiamine for 12 or 18 hours to induce expression from the *nmt1* promoter (during the last step, cells were further diluted as necessary to keep them in the exponential phase of growth). Cells were harvested by centrifugation (1000 g, 3 min, room temperature), suspended in TES buffer (10 mM Tris pH 7.5, 10 mM EDTA pH 8, 0.5% SDS), and total RNA was isolated by phenol-chloroform extraction followed by sodium acetate/ethanol precipitation and column purification using the RNeasy Mini kit (Qiagen).

Five to twenty micrograms of total RNA were labelled by directly incorporating either Cy3- or Cy5-dCTP, or Alexa Fluor 555- or 647-aha-dUTP through reverse transcription and used to hybridize either onto in-house glass DNA microarrays containing probes for >99% of all known and predicted fission yeast genes as described [37], or onto Agilent 8x15K or 4x44K custom-made *S*. *pombe* expression microarrays according to the manufacturer’s protocols. The microarrays were scanned using a GenePix 4000 Laser Scanner (Molecular Devices) and signals were extracted with GenePix Pro 6.0 (Molecular Devices). Initial data processing and normalization were performed using an in-house script [37]. GeneSpring GX (Agilent) and R were used for data evaluation.

The reported values represent gene expression levels for each experimental condition relative to the expression levels in an untreated wild-type or vector-only control from the same experiment. At least two independent biological repeats with dye swap were performed for each experiment (S4 Table). Differentially expressed genes (DEGs) were called based on a conservative fixed cut-off of 2-fold change vs expression levels in the corresponding control sample. We only considered genes with consistent changes across biological repeats (in 2/2 cases, 2/3 cases and 3/4 cases, respectively, depending on the number of repeats available; see S4 Table for details). The significance of overlaps between DEG lists from various conditions was determined by Fisher's exact test with Bonferroni correction for multiple testing in R. For clustering analysis, only those DEGs for which data were available from at least 60% of microarray experiments were used. Clustering was performed based on Euclidean distances using the ‘complete linkage’ method in R. Five major clusters were obtained by this approach. Upon visual inspection of the algorithmic clustering, Cluster 5 was then manually subdivided into Clusters 5a and 5b. AnGeLi, an in-house software tool employing Fisher's exact test with Bonferroni correction (0.05 significance level), was used for functional enrichment analyses of the microarray results (www.bahlerlab.info/AnGeLi; Bitton *et al*., in preparation). Microarray data are available in the ArrayExpress database (www.ebi.ac.uk/arrayexpress) under accession number E-MTAB-2724.

### Chromatin immunoprecipitation and deep sequencing (ChIP-seq)

The protocol for chromatin immunoprecipitation followed by deep sequencing was adapted from [38]. Five hundred millilitre cultures of strains expressing TAP-tagged CSL proteins and of an untagged strain were grown in YES to the density of 1 × 10^7^ cells/ml, fixed with 1% formaldehyde for 30 min, quenched with 125 mM glycine, washed with PBS and broken with glass beads in Lysis Buffer (50 mM Hepes pH 7.6, 1 mM EDTA pH 8.0, 150 mM NaCl, 1% Triton X-100, 0.1% sodium deoxycholate, FY protease inhibitors [Serva]) using the FastPrep24 machine (MP Biomedicals). Extracted chromatin was sheared with the Bioruptor sonicator (Diagenode) to yield DNA fragments of ~200 bp. DNA quality and quantity was checked by Bioanalyzer (Agilent). An aliquot of input DNA was kept, and five milligrams of chromatin extract were used for immunoprecipitation with magnetic IgG beads (1:1 mixture of cat. nos. 112.03D and 110.41, Invitrogen). The precipitated material was washed twice with Lysis Buffer (see above), Lysis 500 Buffer (50 mM Hepes pH 7.6, 1 mM EDTA pH 8.0, 500 mM NaCl, 1% Triton X-100, 0.1% sodium deoxycholate), LiCl/NP-40 Buffer (10 mM Tris-HCl pH 8.0, 1 mM EDTA pH 8.0, 250 mM LiCl, 1% Nonidet P-40, 1% sodium deoxycholate), once in TE (10 mM Tris-HCl pH 8.0, 1 mM EDTA), and eluted in Elution Buffer (50 mM Tris-HCl pH 8.0, 10 mM EDTA, 1% SDS). Cross-links were reversed overnight at 60°C, samples were treated with DNase-free RNase (Roche) followed by proteinase K (Invitrogen), and purified using phenol-chloroform extraction and sodium acetate/ethanol precipitation. Two independent biological repeats were performed.

To construct sequencing libraries, DNA fragments were end-repaired using the End-it kit (Epicentre Biotechnologies), purified using the DNA Clean&Concentrator-5 kit (Zymo Research), deoxyadenine-tailed using the 3'-5' exo- Klenow fragment (New England Biolabs), purified again, sequencing adapters from the Illumina Multiplexed PE Sample Preparation Kit were ligated to the DNA fragments using the Quick T4 DNA Ligase (New England Biolabs), samples were purified again, and sequencing libraries were amplified using the appropriate primers from the Illumina Multiplexed PE Sample Preparation Kit. Finally, libraries were purified using the AMPure XP beads (Agencourt), pooled and sequenced on an Illumina Hi-Seq machine (6-fold multiplexing per lane) at the Genomics Core Facility, CR-UK Cambridge Research Institute, UK.

Sequencing reads were de-multiplexed, aligned to the reference fission yeast genome (release 12) using BWA 0.6.1 [39], processed with the samtools 0.1.18 package [40], and inspected using IGV browser 2.0.23 and the igvtools package [41]. Statistically significant peaks of ChIP-seq signals (p ≤ 1× 10^−5^) were called against IP data from an untagged strain using MACS 1.4.1 [42] (when calling ChIP-seq peaks against the respective input DNA data, MACS reported an excess of false positives, likely because the input DNA libraries contained ~10 times more reads than the IP libraries); only peaks identified in both biological repeats were used in subsequent analyses. Potential binding motifs were searched for within 100 bp under the ChIP-seq peak summits with MEME 4.8.1 [43].

ChIP-seq data are available in the ArrayExpress database (www.ebi.ac.uk/arrayexpress) under accession number E-MTAB-2725.

For conventional ChIP-qPCR experiments, 50 ml cultures were used and the immunoprecipitated DNA was purified using 10% Chelex 100 resin (Bio-Rad). For HA-tagged strains, 5 μg of anti-HA tag rabbit polyclonal antibody (ab9110, Abcam; Antibody Registry acc. no. AB_307019) and protein A sepharose beads (GE Healthcare) were used for precipitation. Quantitative PCR was performed using the MESA GREEN qPCR MasterMix Plus for SYBR (Eurogentec) and the LightCycler 480 II instrument (Roche). The primers used are listed in S2 Table.

### Electrophoretic mobility shift assay (EMSA)

The analysis of DNA binding by Cbf11 was described in detail previously [22]. Briefly, cells were harvested at the density of 2.5 × 10^7^ cells/ml by centrifugation, washed with STOP buffer (150 mM NaCl, 50 mM NaF, 25 mM HEPES, 1 mM NaN_3_; pH 8) and kept at -80°C. Native extracts were prepared in Lysis/Gelshift Buffer (25 mM HEPES, 0.1 mM EDTA, 150 mM KCl, 0.1% Triton X100, 25% glycerol, 1M urea, 2 mM DTT, FY protease inhibitors [Serva]; pH 7.6) by breaking the cells with glass beads in a FastPrep24 instrument (MP Biomedicals). Binding to radiolabelled double-stranded DNA probes containing CSL binding sites was detected as slow-migrating bands on a large native 5% polyacrylamide TBE gel. Signal intensities were quantified using the ImageQuant TL 7.0 software (GE Healthcare).

### Western blotting

Proteins were separated on a 7.5% Tris-glycine-SDS gel, transferred to a nitrocellulose membrane and probed with either a rabbit polyclonal anti-TAP (CAB1001, Open Biosystems; 1:1000 dilution; Antibody Registry acc. no. AB_10709700) or mouse monoclonal anti-PSTAIRE antibody (anti-Cdc2 loading control; P7962, Sigma; 1:8000 dilution; Antibody Registry acc. no. AB_261183), as appropriate. A goat-anti-rabbit or goat-anti-mouse alkaline phosphatase-conjugated secondary antibody (170–6518, 170–6520, Bio-Rad; 1:2000 dilution; Antibody Registry acc. nos. AB_11125338, AB_11125348) were used as required.

### Flow cytometry

Cells were fixed with 70% ethanol, rehydrated in 50 mM sodium citrate, treated with RNase and stained with propidium iodide (4 μg/ml) as described [44]. Highly flocculating strains were treated with 20 mM EDTA and mildly sonicated to reduce cell clumping. DNA content was measured using a BD LSR II instrument (BD Biosciences) or CyAn ADP Analyzer (Beckman Coulter); at least 10,000 cells were measured for each sample. Data were analysed in WinMDI 2.9 (The Scripps Research Institute, San Diego, CA, USA) and FlowJo (FlowJo, LLC). Gating of singlet cells was performed as described [45] where required.

## Results and Discussion

### Antagonism between Cbf11 and Cbf12 at transcriptome level

Cbf11 and Cbf12 bind to DNA in a sequence-specific manner *in vitro* [21,22], and also *in vivo* [23,24]. Furthermore, both CSL proteins can drive gene expression from a CSL response element-containing minimal promoter on a plasmid-based reporter system *in vivo* [23]. Thus, as in Metazoa [15], Cbf11 and Cbf12 likely function as transcription factors [23]. We therefore applied expression microarrays to determine which genes are potentially regulated by CSL proteins, and whether altered gene expression can explain the mutant phenotypes associated with CSL gene manipulation [22].

**Fig 1:**
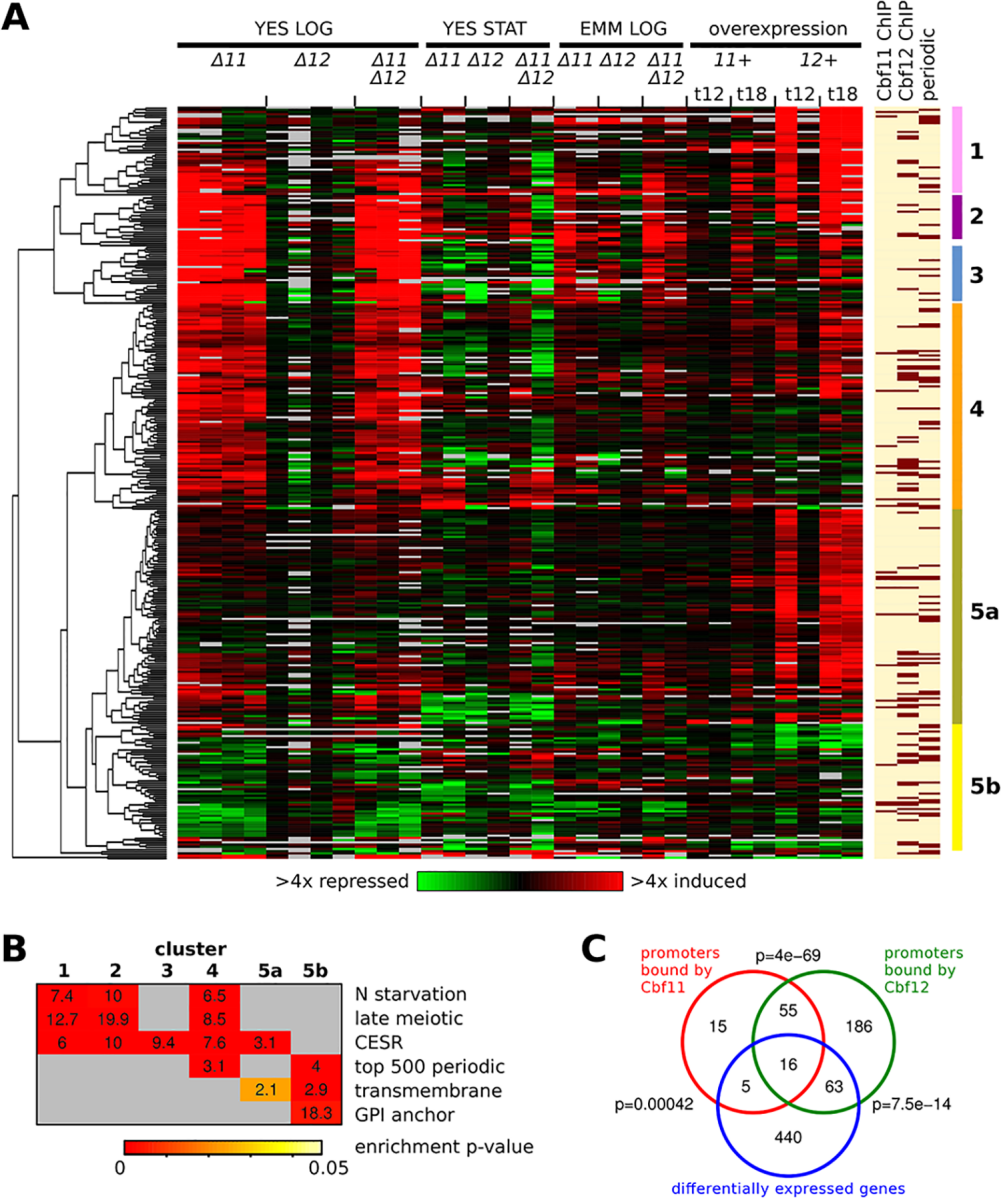
Genes regulated by Cbf11 and Cbf12. **(A)** Heatmap of expression ratios of DEGs in CSL knock-outs under several conditions (‘LOG’–exponential growth; ‘STAT’–stationary phase) and during CSL overexpression (‘OE’; 12 or 18 hours post induction). Different data columns under the same condition represent independent biological repeats. The mRNA levels at each condition relative to the levels in wild-type control cells are colour-coded as indicated at bottom, with missing data in grey. CSL binding to the upstream intergenic regions of the respective DEGs, as detected by ChIP-seq from cells growing exponentially in YES, and periodicity of gene expression during cell cycle [47] are indicated at right (dark bars signify CSL binding/periodic expression). Six major DEG clusters (‘1’-‘5a/b’) are indicated by the right-most colour bars. **(B)** Functional enrichment analysis of the DEG clusters from (A). Numbers in the matrix represent fold enrichment in the cluster compared to all other genes, and the enrichment significance is denoted by the colour of the cell background. ‘N starvation’–genes induced upon nitrogen removal [48]; ‘late meiotic’–genes induced after meiotic divisions [48]; ‘CESR’–core environmental stress response genes [49]; ‘top 500 periodic’–top-ranking 500 genes expressed periodically during cell cycle [47]; ‘transmembrane’–genes coding for transmembrane proteins [50]; ‘GPI anchor’–genes coding for GPI-anchored proteins [51]. **(C)** Venn diagrams showing overlaps between all CSL DEGs and genes with CSL binding in their upstream intergenic regions, as detected by ChIP-seq from cells growing exponentially in YES. The p-values (one-sided Fisher's exact test) for significance of overlap are indicated.

We analysed the transcriptomes of *Δcbf11* and *Δcbf12* single and double deletion mutants under various growth conditions (S4 Table), comprising rapid proliferation in rich YES or minimal EMM media, and entry into stationary phase (YES), where cells cease to proliferate. Transcription factor overexpression, called ‘phenotypic activation’, can be powerful for identifying target genes [46]. We therefore also assayed cells during strong *cbf11* and *cbf12* overexpression (12 and 18 hours post induction) in EMM. Differentially expressed genes (DEGs) in CSL-manipulated cells relative to wild-type cells were determined for each experimental condition based on a 2-fold cut-off, resulting in 524 DEGs overall (S5 Table). Filtering and hierarchical clustering revealed 6 major clusters, comprising 340 genes that showed differential expression in CSL-manipulated strains under at least one condition tested (Fig 1A and S6 Table).

We then performed functional enrichment analyses in these gene clusters (Fig 1B). Clusters 1–5a were significantly enriched for core environmental stress response (CESR) genes, which are induced or repressed under various stress conditions, primarily by the mitogen-activated protein (MAP) kinase Sty1^p38^ and its downstream effector, the transcription factor Atf1 [49]. In addition, Clusters 1, 2 and 4 were also enriched for genes induced during nitrogen starvation, and late during sexual differentiation (‘late meiotic’ genes) [48]. The latter two gene lists largely overlap with the CESR genes. The significance of CESR gene deregulation in *Δcbf11* and *cbf12-*overexpressing cells is discussed below. Notably, Clusters 4 and 5b were also enriched for genes showing periodic expression during the cell cycle [47]. The deregulation of these genes is consistent with the CSL cell-cycle phenotypes described previously [22]. Finally, genes coding for transmembrane and GPI-anchored proteins were enriched in Clusters 5a and 5b, suggesting that their deregulation underlies the altered cell adhesion in CSL-manipulated cells [22,24]. Furthermore, GPI-anchored proteins have been implicated in cell shape specification in fission yeast [26] and their aberrant expression might therefore contribute to the cell morphology defects o*f Δcbf11* cells [22].

We conclude the following from these results:

1. Only few DEGs (<10) were evident in the transcriptomes of *Δcbf12* cells under any condition studied (S1 Fig, panel A). This finding is consistent with the scarcity of mutant phenotypes associated with *Δcbf12* cells under standard growth conditions [22].
2. For *Δcbf11* cells, by far the highest number of DEGs was identified during rapid growth in YES (125/5 genes up/downregulated; S1 Fig, panel A). Both early stationary phase in YES and growth in EMM yielded much fewer DEGs (23/19 and 17/4 genes up/downregulated, respectively), and they largely overlapped with DEGs from *Δcbf11* cells growing rapidly in YES. These findings suggest that Cbf11 controls more genes during rapid cell proliferation (see below).
3. The lists of DEGs from *Δcbf11* single and *Δcbf11 Δcbf12* double-mutant cells largely overlapped (S1 Fig, panel A). For example, from the 125 genes upregulated in *Δcbf11* cells rapidly growing in YES, 112 were also found among the 164 genes upregulated in the CSL double mutant under the same condition. This result is in accordance with the double mutant resembling the phenotypes of the *Δcbf11* single mutant in most aspects, except for abnormal cell adhesion (reduced cell adhesion in both *Δcbf12* single and *Δcbf11 Δcbf12* double mutant) [22].
4. While strong overexpression of *cbf11* resulted in only small transcriptome changes (16/2 genes up/downregulated at 18 hours post induction; S1 Fig, panel A), overexpression of *cbf12* had a much more pronounced effect, with 282 genes upregulated and 17 genes downregulated at 18 hours post induction. Again, this result is consistent with our previous work where medium-strength overexpression of *cbf11* resulted in no apparent cell phenotypes while overexpression of *cbf12* was toxic [22].
5. Genes upregulated in *Δcbf11* cells growing rapidly in YES significantly overlapped with genes upregulated after 18 hours of *cbf12* overexpression (S1 Fig, panel A; 34 genes, p = 3.8 × 10^−17^). Previous analysis of phenotypes associated with *cbf11* deletion and *cbf12* overexpression indicated that Cbf11 and Cbf12 function in an antagonistic manner [22]. The microarray data now reveal that this is also true at the level of transcription regulation. Furthermore, the number of genes affected by Cbf11 and Cbf12 in opposite directions is likely an underestimation due to the stringent cut-offs used for DEG calling. When the average expression of each gene per experimental condition was plotted for all individual DEG clusters, a clear antagonistic trend in regulation by the two CSL paralogs was evident (S1 Fig, panel B). This antagonism between Cbf11 and Cbf12 is likely physiologically relevant since as little as ~4-fold *cbf12* mRNA upregulation, which occurs under some growth conditions [22], triggered *Δcbf11*-like phenotypes [27].

### Cbf11 and Cbf12 bind to overlapping set of genomic loci *in vivo*

Expression microarrays provide only limited insight to distinguish direct from indirect regulation of gene expression. To identify direct CSL target genes, we performed chromatin immunoprecipitation followed by deep sequencing (ChIP-seq) for TAP-tagged Cbf11 and Cbf12 from cells grown exponentially in YES–one of the conditions with the highest number of CSL DEGs. We found 121 (for Cbf11) and 380 (for Cbf12) genes with significant and reproducible peaks of ChIP-seq coverage in their upstream intergenic regions, where promoters are likely located (Fig 1A and 1C). The amplitude of the ChIP-seq peaks was generally lower for Cbf11 in both biological repeats, increasing the probability of missing some binding events. A selection of CSL ChIP-seq peaks was validated by ChIP followed by quantitative PCR (qPCR), using strains with TAP-tagged or HA-tagged Cbf11 and Cbf12. The two ChIP methods showed good agreement (S2 Fig).

The overlap between the CSL ChIP-seq targets (91 and 320 genes for Cbf11 and Cbf12, respectively, for which expression data were available in our microarray dataset) and the overall 524 DEGs identified by microarrays (which include the DEGs filtered out for clustering analysis) was limited: only 21 genes (~23% of ChIP-seq targets) for Cbf11 and 79 genes (~25% of ChIP-seq targets) for Cbf12, yet these overlaps were statistically significant (Fig 1C). This finding suggests that most changes in transcript levels observed upon CSL manipulation could have been brought about indirectly, perhaps through a regulatory cascade as in the case of metazoan CSL family members [52]. Intriguingly, the putative direct CSL targets included several genes coding for characterized or predicted regulators [50], such as transcription factors (Rsv2, SPAC2H10.01, Gsf1, SPCC1393.08), kinases and phosphatases (Byr2, Srk1, Oca2, Pyp2), and RNA-binding proteins (Mei2, Mug24), some of which with known roles in cell-cycle regulation [53–55]. It is possible that additional direct CSL targets could be identified using ChIP-seq under different growth conditions. Also, some CSL binding events detected in cells growing exponentially in YES might only result in gene expression regulation upon a specific stimulus that was not tested in this study. Another potential explanation for the limited overlap between CSL ChIP-seq targets and DEGs is the stringency we applied for DEG calling. Indeed, Cbf11-bound genes tend to be more differentially regulated in *Δcbf11* cells growing exponentially in YES compared to non-bound genes, and significant differential regulation occurs also for the set of Cbf12-bound genes under *cbf12* overexpression (S3 Fig).

The ChIP-seq targets of Cbf11 and Cbf12, on the other hand, showed a highly significant overlap (71 shared genes, p = 4 × 10^−69^). However, most of these common CSL-bound genes did not show differential expression upon CSL manipulation (Fig 1C). It is possible that the regulatory crosstalk between the two CSL paralogs is mediated through a mechanism that does not involve direct binding of Cbf11 and Cbf12 to the same, antagonistically regulated promoters; however, 16 out of the 21 Cbf11-bound DEGs (~76%; mostly from Clusters 4 and 5a) were also bound by Cbf12 (Fig 1C). The detailed mechanism of CSL cross-regulation thus remains to be elucidated by future experiments.

Chua and colleagues previously reported expression microarray data for *cbf11* deletion (YES medium) and *cbf12* overexpression (EMM medium), and ChIP-chip data for ectopically overexpressed Cbf12 (EMM medium) [24]. There is significant overlap between the Chua dataset and the corresponding subset of our results (S4 Fig). However, Chua and colleagues typically report many more target genes. There are several possible reasons for this discrepancy. First, we performed ChIP-seq using tagged endogenous CSL alleles expressed close to physiological levels, as opposed to ectopic overexpression employed by Chua. Second, we conducted more biological repeats of both transcriptomic and ChIP analyses and applied stringent cut-offs, limiting the number of reported target genes. Third, different genomic platforms were used in the two studies. Thus, we believe that our data validate, and further refine the CSL target genes reported by Chua *et al*. [24].

We conclude that Cbf11 and Cbf12 bind to an overlapping set of target genes, including genes with important roles in cell cycle regulation, while the detected overlap with genes showing altered expression in CSL-manipulated cells is much more limited.

### Subset of cell cycle-regulated genes is deregulated upon CSL manipulation

**Fig 2:**
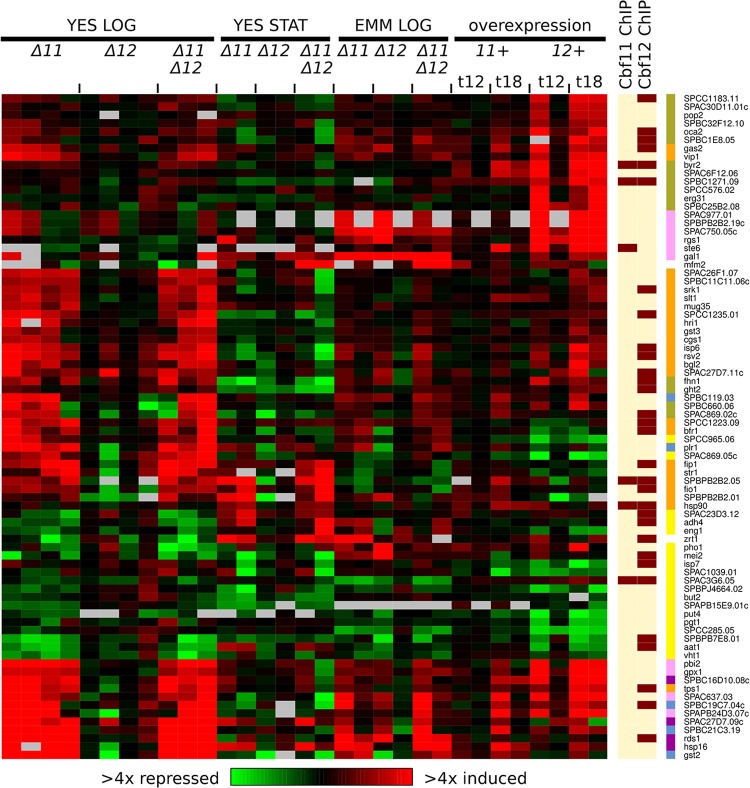
Subset of cell cycle-regulated genes show altered expression upon CSL manipulation. Heatmap of expression ratios of DEGs (as in Fig 1A) that belong to top-ranking 500 genes expressed periodically during the cell cycle [47]. CSL binding to promoters of respective DEGs, as detected by ChIP-seq from cells growing exponentially in YES, is indicated at right (dark bars signify CSL binding). Cluster membership is indicated by right-most colour bars (colour coding as in Fig 1A).

The CSL DEG clusters 4 and 5b were enriched for genes expressed periodically with the fission yeast cell cycle [47] (Fig 1B). Notably, similar enrichment was also found in the 34 DEGs commonly upregulated in both *Δcbf11* and *cbf12-*overexpressing cells (11 periodic genes, p = 0.02). Overall, 81 periodic genes were either induced or repressed in response to altered CSL levels (Fig 2 and S7 Table). The distribution of peak expression times of these genes during the wild-type cell cycle shows only slight enrichment around the S/G2 transition, compared to all periodically expressed genes (S5 Fig); no further cell-cycle phase-specific enrichment was detected by separate analyses of periodic genes up/downregulated in *Δcbf11* cells grown exponentially in YES.

Interestingly, for 31 of these 81 periodic genes (~38%), Cbf12 binding to the corresponding upstream intergenic regions was detected by ChIP-seq, while the overall overlap of CSL DEGs with Cbf12 binding sites was just ~25%. Previous genome-wide studies identified novel DNA motifs possibly involved in the regulation of periodic transcription [2–4]; however, none of these motifs is related to the CSL response element sequence [22,56,57]. Cbf11 binding was only detected at the promoters of 6 of the 81 periodic CSL DEGs.

While our expression microarray and ChIP-seq data were obtained using unsynchronized cell populations, prohibiting direct assessment of CSL role in the temporal control of gene expression, they nevertheless provide useful indications which genes and processes are affected by CSL proteins during the cell cycle:

One of these periodic DEGs is *eng1*, encoding an endo-1,3-beta-glucanase required for primary cell septum disassembly, which was downregulated 1.5-fold in *Δcbf11* cells and 2.2-fold in *cbf12-*overexpressing cells. Absence of Eng1 results in the inability of daughter cells to separate after cytokinesis [8], reminiscent of the cell separation phenotype observed upon CSL manipulation [22].

**Fig 3:**
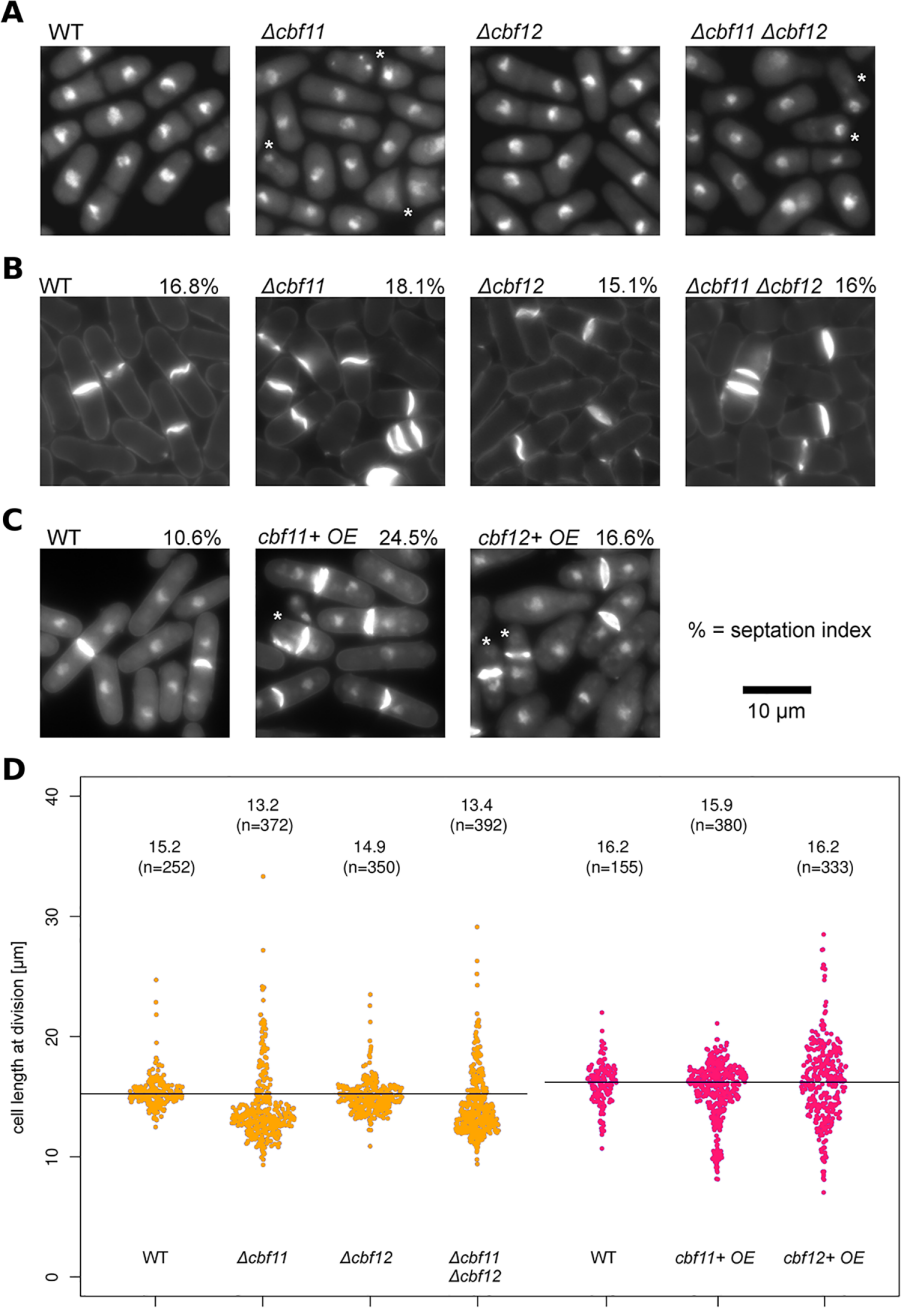
Cbf11 and Cbf12 affect multiple aspects of cell division cycle. **(A)** CSL knock-out cells grown in YES were fixed and stained with DAPI (nuclei). Overall, 10–20% cells lacking *cbf11* underwent catastrophic mitosis (‘cut’ phenotype, denoted by asterisks). **(B)** CSL knock-out cells (YES medium) or **(C)** CSL-overexpressing cells (EMM medium) were fixed and stained with calcofluor (B; septum and cell wall) or calcofluor and DAPI (C; septum, cell wall and nucleus). Note the multiple septa in *Δcbf11* and *Δcbf11 Δcbf12* cells (B), and septum formation in absence of nuclear division in cells overexpressing *cbf11* or *cbf12* (‘cut’ phenotype; asterisks) (C). The fractions of dividing cells (septation index; %) were increased upon *cbf11* and *cbf12* overexpression (*n* >1000 cells). **(D)** The length of fully septated cells from (B, C) was measured (orange, red). Each dot represents a single cell measurement; median length and *n* values are indicated above each distribution; WT/control median values are indicated as black horizontal lines. Deletion of *cbf11* resulted in decreased cell length at division. Severely shortened cell length at division was observed in a fraction of cells overexpressing *cbf11*. Cells overexpressing *cbf12* displayed gross deregulation of cell size at division.

Other periodic DEGs include *cgs1*, encoding the regulatory subunit of protein kinase A, which was upregulated on average 2-fold in *Δcbf11* cells and 1.7-fold in *cbf12-*overexpressing cells. In low glucose, Cgs1 binds to and inhibits Pka1, the catalytic subunit of PKA, which regulates the cell cycle in response to nutrients [58]. Notably, cells lacking PKA activity are small [26,59]. Another example is *srk1*, which was upregulated 2.4-fold in *Δcbf11* cells and 1.9-fold in *cbf12-*overexpressing cells, and its promoter is bound by Cbf12 (Fig 2). Srk1 is a MAP kinase-activated protein kinase that acts downstream of the Sty1 MAP kinase and is involved in negative regulation of G2/M transition of the mitotic cell cycle. Cells overexpressing *srk1* are long [53]. Such overexpression of proteins with important regulatory effects on cell-cycle progression might well contribute to the cell-cycle phenotypes observed in cells lacking Cbf11 or overproducing Cbf12 ([22] and Fig 3).

Additional experiments are needed to determine any direct contributions of Cbf11 and Cbf12 towards regulating the expression of periodically expressed genes, some of which can explain the cell-cycle progression and cellular morphology defects associated with altered CSL protein levels.

### Cbf11 and Cbf12 affect specific aspects of cell-cycle progression

Initial characterization of Cbf11 and Cbf12 revealed that cells with altered CSL levels (*cbf11* deletion, *cbf12* overexpression) display defects in septation and impaired coordination of cell and nuclear division, resulting in catastrophic mitosis and the ‘cut’ phenotype (Fig 3A and 3B and [22]; typically 10–20% of *Δcbf11* cells are ‘cut’). The previously reported cell cycle-related defects triggered by the overexpression of *cbf12* were observed in cells grown in the MB medium [22], which is nutrient-poor compared to the more widely used EMM. Also, overexpression driven by the inducible medium-strength *nmt1* promoter variant used previously might not have been sufficient to trigger a full phenotypic response to increased gene dosage. Therefore, we analysed cultures grown in EMM where Cbf11 or Cbf12 were overexpressed from the full-strength *nmt1* promoter. At 17 hours post induction, the ‘cut’ phenotype was detected both in cells overexpressing *cbf12* (~5% ‘cut’) and in cells overexpressing *cbf11* (~6% ‘cut’; Fig 3C).

Prompted by the discovery of deregulated expression of some periodic genes in CSL mutants (Fig 2), we tested whether other aspects of cell-cycle control are also affected by CSL manipulation. Increased or decreased cell lengths at division are hallmarks of mutations in important cell-cycle regulators such as *cdc2* ^CDK1^, *wee1*, or *cdc25* [26]. We measured the cell length at division in wild-type and CSL single and double mutant strains growing exponentially in YES medium, and in CSL-overexpressing cells growing in EMM (Fig 3D). Deletion of *cbf11*, either alone or in combination with deletion of *cbf12*, typically resulted in decreased maximum length of septated cells (‘wee’ phenotype [26]). In addition, a minor subpopulation of *Δcbf11* cells showed increased length of septated cells compared to wild type. Furthermore, a subpopulation of *cbf11*-overexpressing cells displayed severely reduced maximum cell length. These results point to a possible role for Cbf11 in the timing of the G2/M transition. While no change in septated cell length was detected in *Δcbf12* cells, there was gross deregulation of maximum cell length upon *cbf12* overexpression (Fig 3D). Cell size alteration upon *cbf12* overexpression has also been reported by others [25].

The timing of cell-cycle events also manifests in the percentage of cells dividing in an unsynchronized population at any given time (septation index; Fig 3B and 3C). While neither CSL deletion mutant showed any marked changes in the septation index, it was increased in both *cbf11-* and *cbf12*-overexpressing cells (24.5% and 16.6%, respectively) compared to wild type (10.6%).

Taken together, these findings indicate important roles for CSL proteins in the cell's decision to divide, and support our previous notion that balanced Cbf11 and Cbf12 activities are required for proper cell-cycle progression [22]. Notably, pleiotropic cell cycle and morphology phenotypes, similar in several aspects to *Δcbf11* cells (Fig 3 and [22]), have also been observed when the *fkh2* transcription factor gene, which regulates M-phase genes, was deleted or overexpressed [9,10].

### *cbf11* interacts genetically with *sty1* and *pka1* pathways

**Fig 4:**
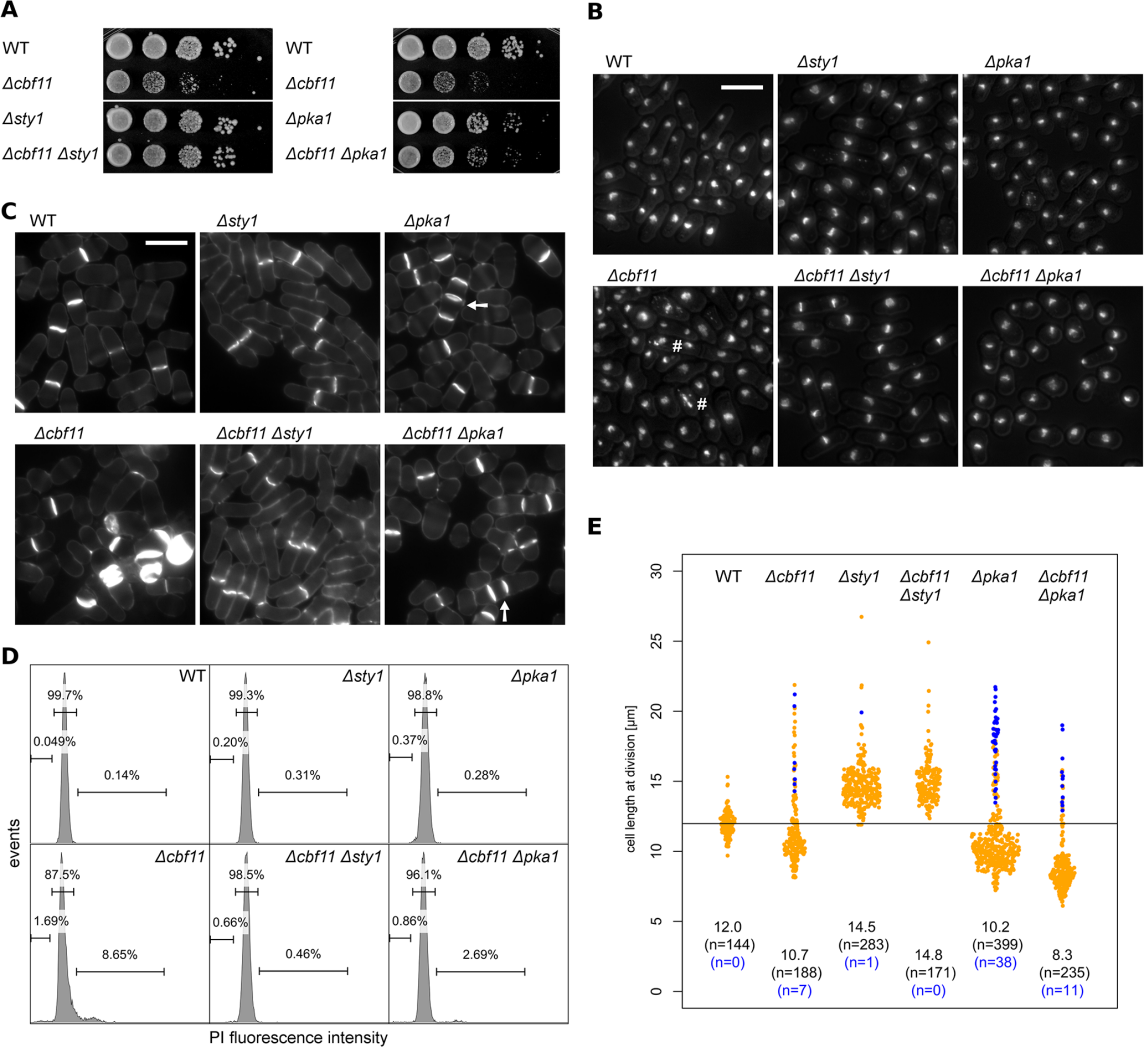
*cbf11* interacts genetically with *sty1* and *pka1* pathways. **(A)** 10-fold serial dilutions of cultures with the indicated genotypes were spotted on YES plates and grown for 2 days. The slow growth phenotype of *Δcbf11* cells is strongly and moderately suppressed by the deletion of *sty1* and *pka1*, respectively. **(B)** Cells growing exponentially in YES were fixed and stained with DAPI. Nuclear integrity defects of *Δcbf11* cells (marked with '#') are diminished by deletion of *sty1* or *pka1*. The DAPI signal was overlaid with the corresponding DIC image to visualize cell contours. Scale bar 10 μm. **(C)** Calcofluor staining documents that the occurrence of *Δcbf11*-associated septation defects (e.g., single cells with multiple septa) is decreased in the double mutants with *sty1* and *pka1*. Multicellular filaments are marked by arrows. Scale bar 10 μm. **(D)** Flow cytometry analysis of DNA content in fixed, propidium iodide-stained cells grown to the exponential phase in YES. Deletion of *cbf11* results in aberrant DNA content distribution, which is corrected by deletion of *sty1* and, in part, *pka1*. Fractions of cells with <2C, 2C, and >2C DNA content are indicated in the histograms. **(E)** The length of fully septated cells from (C) was measured. Deletions of both *sty1* and *pka1* have marked influence on the length of *Δcbf11* cells. Each dot represents a single cell measurement; median length and *n* values are indicated above each distribution; WT median value is indicated as a black horizontal line. Data points corresponding to short, multicellular filaments and their *n* values are shown in blue.

As mentioned above, our results (Figs 1 and 2) suggest possible functional links between CSL and two antagonistic systems important for cell-cycle progression–the PKA (Pka1) and stress-activated MAP kinase (Sty1) pathways [60]. Therefore we constructed and characterized double mutants of *Δcbf11* with *Δpka1*/*Δsty1* to assess any genetic interactions related to cell-cycle control. As shown in Fig 4A, deletion of *sty1* strongly suppressed the growth defect of *Δcbf11* cells; moderate suppression was also evident in *Δcbf11 Δpka1* cells. Microscopic analysis of these double mutants revealed diminished *Δcbf11*-associated defects in nuclear integrity and septum formation (Fig 4B and 4C); this was also confirmed by FACS analysis of DNA content (Fig 4D). Furthermore, a fraction of septated *Δcbf11*, *Δpka1*, and *Δcbf11 Δpka1* cells actually formed multicellular filaments. This phenotype was not additive in the double mutant, indicating common defects in daughter cell separation in *Δcbf11* and *Δpka1* cells (Fig 4C and 4E).

There were also striking effects of *pka1* and *sty1* mutations on cell size at division in *Δcbf11* cells (Fig 4E). Sty1 has a role in promoting entry into mitosis and *Δsty1* cells are elongated [61]. Such elongation was uniformly observed in the *Δcbf11 Δsty1* strain. The absence of Sty1 thus completely suppressed the 'wee' phenotype found in most *Δcbf11* cells. Pka1, on the other hand, is a negative regulator of mitotic entry and *Δpka1* cells are short [26,59]⁠, about the same length as most *Δcbf11* cells. Notably, the 'wee' phenotype was further potentiated in *Δcbf11 Δpka1* cells, and the subpopulation of large cells, present in the *Δcbf11* single mutant, was diminished.

Thus, there is indeed crosstalk in the regulation of cell-cycle progression between the CSL transcription factors, and the PKA and stress MAP kinase pathways in fission yeast. Since many Sty1 target genes are upregulated in *Δcbf11* cells (Fig 1A and 1B), Cbf11 might act as a negative regulator of Sty1.

### Defects of *Δcbf11* cells are diminished in minimal medium

**Fig 5:**
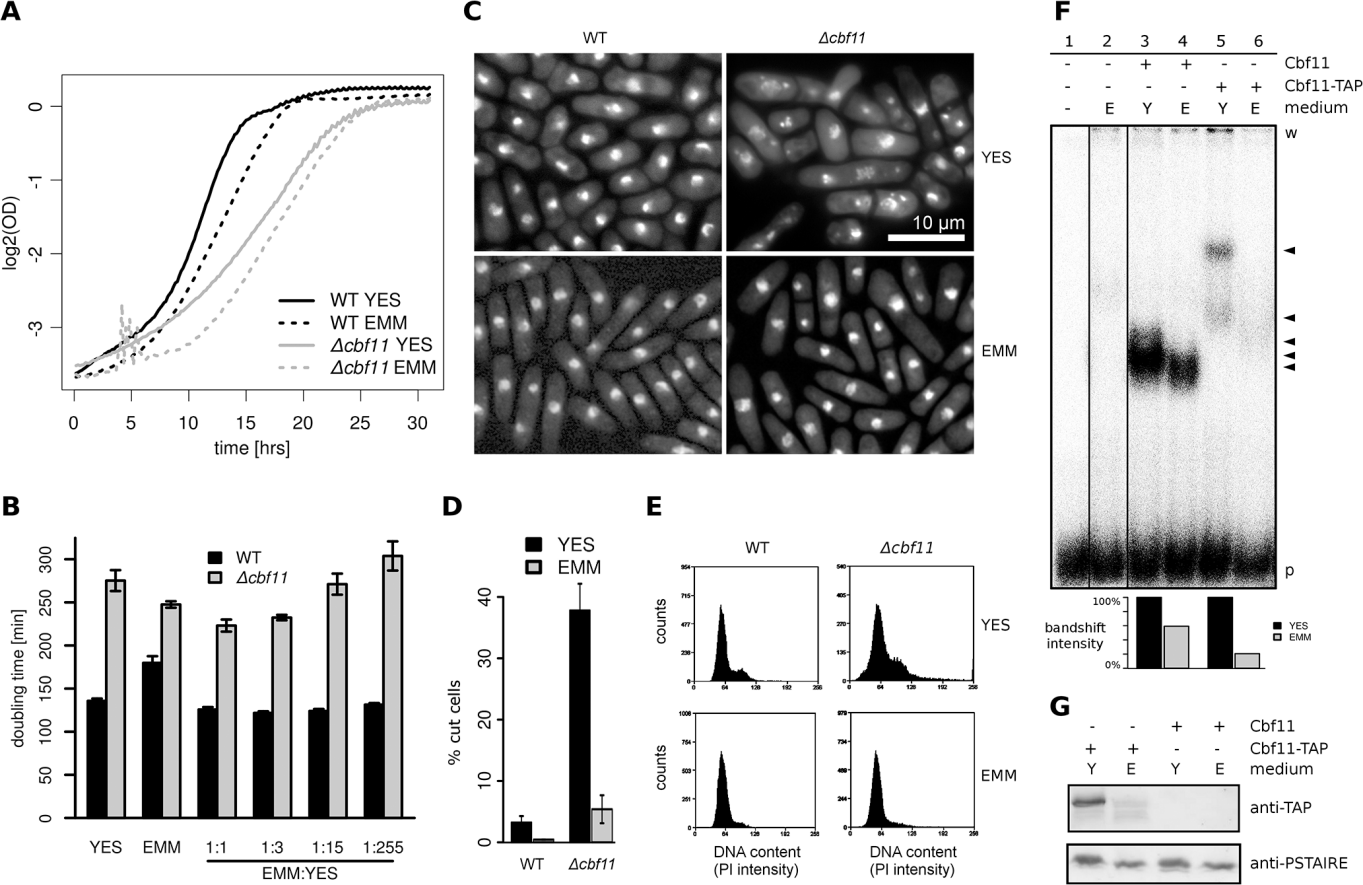
Cbf11 protein expression and defects of *Δcbf11* cells are diminished in minimal medium. **(A)** Growth curves of WT and *Δcbf11* cultures in different liquid media show that the slow growth of *Δcbf11* cells observed in YES is partially suppressed in EMM (i.e., curve slope is increased in EMM). The noise appearing during the *Δcbf11* lag phase in EMM was caused by cell flocculation. **(B)** Doubling times of exponentially growing WT and *Δcbf11* cultures in the indicated media. The addition of EMM to YES causes dose-dependent decrease in doubling time of *Δcbf11* cells. **(C)** Microscopy of fixed, DAPI-stained wild-type and *Δcbf11* cells grown to exponential phase in YES or EMM media. Cells lacking *cbf11* display heterogeneous morphology, cell separation defects and the ‘cut’ phenotype when grown in YES. These mutant phenotypes are largely absent from cells grown in EMM. **(D)** Quantification of the occurrence of the ‘cut’ phenotype in cells from panel (C). Mean values ± SD from three independent biological repeats are shown (*n* > 200 cells). **(E)** Flow cytometry analysis of DNA content in wild-type and *Δcbf11* cells growing exponentially in YES or EMM. The broad signal distribution in *Δcbf11* cells from rich medium is narrowed towards wild-type values when cells were grown in EMM (*n* > 15,000 cells). **(F)** EMSA assay of fission yeast cell extracts. When cells were grown in EMM, the DNA binding activity of Cbf11 decreased by ~40–80% compared to YES. Lane 1: no cell extract added; lane 2: cell extract from *Δcbf11 Δcbf12* cells; lanes 3–4: wild-type cells; lanes 5–6, samples from cells expressing a chromosomally TAP-tagged version of Cbf11. ‘w’ and ‘p’ denote the position of wells and free probe, respectively. The arrowheads mark bands corresponding to the DNA binding activity of Cbf11. The bar chart at bottom shows the quantification of bandshift intensities in the respective lanes. Irrelevant gel lanes were omitted for clarity. **(G)** Western blot detection of Cbf11-TAP in cell extracts from panel F (lanes 5–6, 3–4) showing decreased Cbf11 protein amounts in cells grown in EMM as compared to YES. As a loading control, the blots were probed with an anti-PSTAIRE (Cdc2) antibody. Representative examples of 3 biological repeats are shown in (F, G).

Since both Pka1 and Sty1 pathways are sensitive to nutrient availability [61–64], we tested the influence of growth media composition on *Δcbf11*-associated defects. Wild-type cells grow slower in minimal medium (EMM) than in rich medium (YES). In contrast, the slow growth phenotype of *Δcbf11* cells observed in YES was partially alleviated in EMM (Fig 5A). The *Δcbf11* growth rate showed a further slight increase in a 1:1 mixture of YES and EMM, suggesting that both media contain specific substances (or different concentrations thereof) that are limiting for the growth of *Δcbf11* cells. However, the contribution of EMM seemed to be more important as the *Δcbf11* doubling time increased in a dose-responsive manner with decreasing EMM content in the mixed medium (Fig 5B). Furthermore, the cell morphology and cell separation defects, and the ‘cut’ phenotype observed in *Δcbf11* cells growing exponentially in YES were largely absent in EMM (Fig 5C and 5D). Also, FACS analysis of DNA content confirmed diminished nuclear integrity defects in *Δcbf11* cells grown in EMM (Fig 5E).

Taken together, we propose that Cbf11 is less critical during growth on EMM. This differential requirement is also consistent with the small number of DEGs detected in *Δcbf11* cells grown in EMM (Fig 1A and S1 Fig, panel A). Notably, Cbf11 showed decreased DNA-binding activity *in vitro* (Fig 5F; note that the change in bandshift intensity is less pronounced in wild-type cells compared to Cbf11-tagged cells). This result could reflect lower Cbf11 expression or altered posttranslational modifications of Cbf11 in EMM. Indeed, western blot analysis revealed lower Cbf11 amounts in EMM compared to YES. Furthermore, Cbf11 was present in two isoforms, and their relative quantities were different in YES and EMM (Fig 5G). It remains to be established whether these changes in protein levels and isoforms also occur with untagged Cbf11. Thus, our data suggest that Cbf11 regulates gene expression mainly during rapid cell proliferation in rich medium. The precise nature of the nutrient(s) or other factor(s) critical for Cbf11 activity is currently under investigation.

### Cbf11 regulates a group of lipid metabolism genes

**Fig 6:**
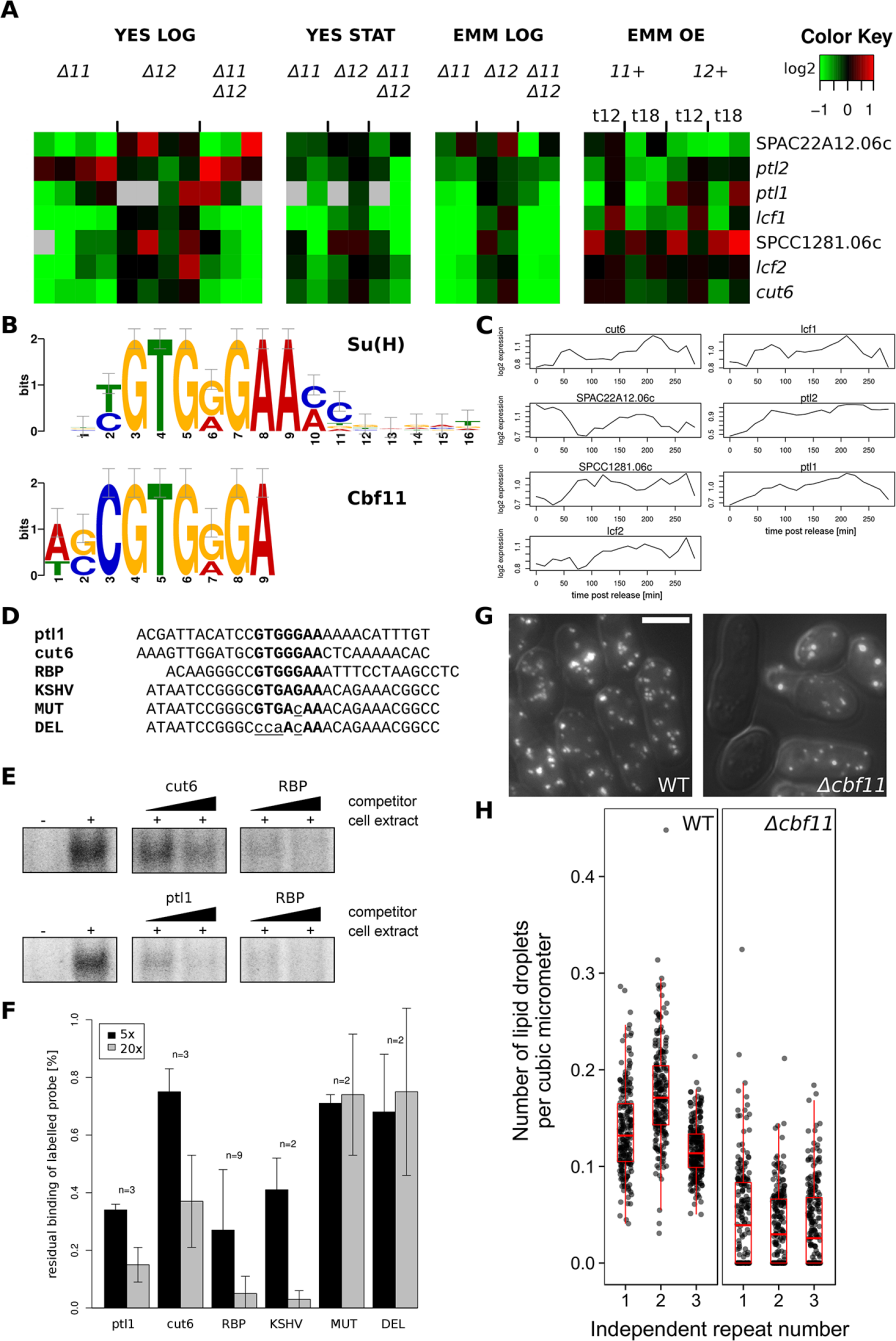
Putative Cbf11-regulated lipid metabolism genes. **(A)** The mRNA levels at each condition relative to the levels in wild-type control cells are colour-coded as indicated at top right, with missing data in grey; for description of cultivation conditions see legend to Fig 1A. **(B)** The sequence motif identified by MEME-ChIP [65] in the Cbf11-bound promoter regions of genes from (A) resembles closely the canonical metazoan CSL response element, ‘Su(H)’. **(C)** Previous transcriptome profiling of the genes from (A) over two cell cycles of elutriation-synchronized wild-type cultures suggest moderate periodic oscillations in the expression of some of these genes. Data taken from [2]. **(D)** Probes used for EMSA experiments (sense strand shown); CSL response elements are in bold and introduced mutations are in lower-case and underlined. ‘RBP’, ‘KSHV’, ‘MUT’ and ‘DEL’ probes were derived from metazoan/viral CSL-responsive genes [22]. **(E)** Representative competitive EMSAs with wild-type cell extracts and radioactively labelled RBP probe; unlabelled *cut6*, *ptl1* and RBP probes were used as competitors (5× and 20× excess). The decrease in band intensity reflects strength of binding of unlabelled competitor probes. **(F)** Quantification of competitive EMSA experiments for 5× and 20× competitor excess. Mean values ± SD are shown. **(G)** Representative examples of live WT and *Δcbf11* cells stained with Nile red to visualize neutral lipid droplets. Scale bar 5 μm. **(H)** Numbers of lipid droplets per cell were normalized to cell volume. Each dot corresponds to one cell (n ≥ 200); data for three independent repeats are shown.

As shown above, the lists of promoters bound by Cbf11 and Cbf12 in unsynchronized cells overlapped significantly, but not fully (Fig 1C). To get further insight into any specific role of Cbf11, we analysed the promoters bound exclusively by Cbf11, which were significantly associated with lipid metabolism genes (7 genes in total, p = 0.019; Table 1 and Fig 6A). Notably, a sequence motif closely resembling the canonical metazoan CSL response element [56,57] was found in the promoters of all these 7 genes (Fig 6B). Furthermore, 6 of these genes were downregulated in *Δcbf11* cells grown exponentially in YES, i.e., under the same conditions as for the ChIP-seq experiment (Fig 6A). Some of these lipid metabolism genes show weakly periodic expression during the cell cycle (Fig 6C, data taken from [2]). We tested whether Cbf11 can bind *in vitro* to double-stranded DNA oligonucleotides containing the *in silico*-identified putative Cbf11 binding sites from *ptl1* and *cut6* promoters. In competitive EMSA assays, Cbf11 indeed bound to these sequences specifically, with somewhat lower affinity compared to the previously tested probes derived from known metazoan CSL binding sites (Fig 6D–6F). We conclude that Cbf11 is likely a direct activator for transcription of lipid metabolism genes.

**Table 1:** Putative Cbf11-regulated lipid metabolism genes.

Gene ID	Name	Description	EC^a^	Periodic^b^
SPAC56E4.04c	*cut6*	acetyl-CoA/biotin carboxylase	6.3.4.14	S; [3]
SPBC18H10.02	*lcf1*	long-chain-fatty-acid-CoA ligase	6.2.1.3	
SPBP4H10.11c	*lcf2*	long-chain-fatty-acid-CoA ligase	6.2.1.3	
SPAC1786.01c	*ptl2*	triacylglycerol lipase	3.1.1.3	S; [3]
SPAC22A12.06c	SPAC22A12.06c	serine hydrolase-like		M; [3]
SPCC1281.06c	SPCC1281.06c	acyl-coA desaturase (predicted)		G2; [3]
SPCC1450.16c	*ptl1*	triacylglycerol lipase	3.1.1.3	

^a^Enzyme Commission number.

^b^Indicates whether a gene has been deemed to be periodically expressed, and the cell-cycle phase of peak expression.

Interestingly, the colonies of the *Δcbf11* strain overproduce a layer of wax-like material on their surface [22], which may result from a possible dysregulation of lipid metabolism when Cbf11 is missing. To assess the impact of *cbf11* deletion on neutral lipid content in cells growing exponentially in YES, we quantified the number of storage lipid droplets using Nile red staining. Compared to wild type, there was a striking depletion of lipid droplets in *Δcbf11* cells, supporting a role of Cbf11 in lipid metabolism. Curiously, in many apparently live *Δcbf11* cells, no lipid droplets were detected at all (Fig 6G and 6H).

Notably, it has been recently reported that mammalian cells remodel their membrane lipid composition during the cell cycle, and these periodic changes are important for proper cell-cycle progression [66]. Importantly, the *cut6* gene is required for proper coordination of cell and nuclear division, and *cut6* mutants show the ‘cut’ phenotype [67], which also occurs in the *Δcbf11* strain [22]. This finding raises the possibility that the ‘cut’ phenotype of *Δcbf11* is mediated by *cut6*.

## Conclusions

CSL transcription factors affect multiple aspects of cell-cycle progression in fission yeast ([22,25] and this study). Our genomic analyses uncovered sets of genes regulated directly or indirectly, and often antagonistically, by Cbf11 and Cbf12, and highlighted specific biological processes in which CSL proteins play a distinct role. We showed that many genes expressed periodically during the cell cycle are deregulated upon CSL deletion/overexpression, which could explain some of the cell-cycle defects associated with CSL manipulation. We further showed that *cbf11* interacts genetically with the nutrient-responsive cell-cycle regulatory pathways controlled by protein kinase A (Pka1) or stress-activated MAP kinase Sty1^p38^. Interestingly, Cbf11 expression is affected by media composition, and Cbf11 activity is required mainly under rapid proliferation in rich medium. Finally, we identified Cbf11 as a novel regulator of lipid metabolism genes. Our results provide a basis for a more detailed understanding of CSL transcription factors and for testing their role in the regulatory relationship between nutrients and cell cycle progression in fission yeast.

## Supporting Information

S1 Fig Comparative analysis of expression microarray data. **(A)** Summary of overlaps of DEG lists for all conditions tested (red label–upregulated genes; green label–downregulated genes). Numbers in matrix represent the numbers of genes shared between the respective DEG lists, and the significance of overlap is denoted by colour of cell background. **(B)** Distributions of DEG expression ratios in individual clusters from Fig 1A. Data from all biological repeats per experimental condition were averaged and plotted. Numbers of DEGs in each cluster are indicated.
(TIF)

S2 Fig Validation of selected CSL binding sites detected by ChIP-seq. Selected CSL binding sites identified from ChIP-seq data by peak calling algorithm MACS [42] were validated by ChIP-qPCR. The ChIP-qPCR enrichment of target loci DNA was normalized to a control locus where no CSL binding was detected by ChIP-seq and plotted against integrated ChIP-seq signal from the region ±100 bp from qPCR amplicon centre (area under peak, AUP).
(TIF)

S3 Fig Differential regulation of CSL-bound genes. **(A)** Average expression values were calculated from all biological replicates of *Δcbf11* cells growing exponentially in YES. Genes were then divided into upregulated (average expression ratio to wild type > 1) and downregulated (average expression ratio to wild type < 1), and further classified by the presence or absence of Cbf11 binding in their promoter. Cbf11-bound genes typically show more pronounced changes in expression compared to other genes. P-values of one-tailed t-test are indicated. **(B)** An analogous analysis as in (A) performed for Cbf12 target genes under *cbf12* overexpression (18 hrs). Again, Cbf12-bound genes typically display more differential expression compared to all other genes.
(TIF)

S4 Fig Comparison of expression microarray and ChIP-seq/chip data for CSL from two studies. Comparison of CSL DEGs and CSL-bound genomic loci between this study (red) and Kwon et al. (green) [24]. Overlap significance was determined using the Fisher's exact test.
(TIF)

S5 Fig Distribution along cell cycle of expression peaks of periodic genes deregulated in CSL mutants. **(A)** Histogram of peak expression times for all top 500 periodic genes (grey) and for the subset thereof that is deregulated in CSL mutants (black). Normalized peak expression times are plotted as the percentage of cell-cycle duration [47]. Cell-cycle phases are indicated on top. **(B)** Cumulative distribution of peak expression times for all top 500 periodic genes (grey). Periodic genes showing deregulation in CSL mutants are highlighted as coloured dots.
(TIF)

S1 Gels Gel images used to create Figs 5F, 5G and 6E. **(A)** EMSA from Fig 5F. Relevant lanes used for Fig 5F are highlighted in red. Blue and yellow rectangles correspond to regions used for densitometry. **(B)** Western blot used for Fig 5G. Relevant lanes used for Fig 5G are highlighted in red. **(C)** EMSAs from Fig 6E. Relevant lanes used for Fig 6E are highlighted in red.
(PDF)

S1 Table Fission yeast strains used in this study. (XLS)

S2 Table Oligonucleotides used in this study. (XLS)

S3 Table Plasmids used in this study. (XLS)

S4 Table Summary of expression microarray experiments performed in this study. (XLS)

S5 Table Genes showing differential expression in CSL deletion or overexpression (from Fig 1A). (XLS)

S6 Table Clusters of genes showing differential expression in CSL deletion or overexpression (from Fig 1A). (XLS)

S7 Table Periodically expressed genes showing differential expression in CSL deletion or overexpression (from Fig 2). (XLS)
