# Correction: Fission Yeast CSL Transcription Factors: Mapping Their Target Genes and Biological Roles

**DOI:** 10.1371/journal.pone.0299200

**Published:** 2024-02-15

**Authors:** Martin Převorovský, Martina Oravcová, Jarmila Tvarůžková, Róbert Zach, Petr Folk, František Půta, Jürg Bähler

After publication of this article [1], the authors discovered that one of the fission yeast strains used in the study had undergone a genome rearrangement, leading to artifactual results.

Here, the authors have provided additional information to clarify these issues:

The supposed *Δcbf11 Δsty1* double deletion strain used in this article contains an ectopic (partial) copy of the *cbf11* gene and, therefore, all experiments and conclusions based on this strain are invalid. The affected results appear in Fig 4 (all panels) of this article, and the invalid conclusion that the deletion of the stress-activated protein kinase Sty1 gene rescues the *Δcbf11*-associated defects is mentioned in the Abstract as well as throughout the Results and Discussion section. All other aspects and conclusions of our article remain valid. It is not possible to perform an analysis of a bona fide *Δcbf11 Δsty1* strain to confirm that *sty1* deletion does not suppress *Δcbf11* because the *Δcbf11 Δsty1* double mutant strain cannot be constructed because there seems to be synthetic lethality [2]. The validity of the other strains used in this article were analyzed and validated.

The authors have provided the following corrections:

- In the fourth sentence of the Methodology/Principal Findings subsection of the Abstract, the correct sentence is: Cbf11 activity is nutrient-dependent and *Δcbf11*-associated defects are mitigated by inactivation of the protein kinase A (Pka1) pathway.
- In the last sentence of the Introduction, the correct sentence is: We further show that the function of Cbf11 in regulating cell-cycle progression is affected by nutrients and by protein kinase A (Pka1) pathway.
- In Results and Discussion, the correct title of the fifth subsection is: “*cbf11* interacts genetically with *pka1* pathway”.
- In the first paragraph of the fifth subsection of the sentence of the Results and Discussion, the correct first three sentences are: As mentioned above, our results (Figs 1 and 2) suggest possible functional links between CSL and the PKA (Pka1) pathway [60]. Therefore we constructed and characterized double mutants of *Δcbf11* with *Δpka1* to assess any genetic interactions related to cell-cycle control. As shown in Fig 4A, moderate suppression was evident in *Δcbf11 Δpka1* cells.
- In the second paragraph of the fifth subsection of the sentence of the Results and Discussion:
  ◦ The correct first sentence is: There were also striking effects of *pka1* mutation on cell size at division in *Δcbf11* cells (Fig 4E).
  ◦ The second, third, and fourth sentences are incorrect and should be ignored.
  ◦ The correct fifth sentence is: Pka1 is a negative regulator of mitotic entry and *Δpka1* cells are short [26,59], about the same length as most *Δcbf11* cells.
- In the third paragraph of the fifth subsection of the sentence of the Results and Discussion:
  ◦ The correct first sentence is: Thus, there is indeed crosstalk in the regulation of cell-cycle progression between the CSL transcription factors, and the PKA pathway in fission yeast.
  ◦ The second sentence is incorrect and should be ignored.
- In Conclusions, the correct fourth sentence is: We further showed that *cbf11* interacts genetically with the nutrient-responsive cell-cycle regulatory pathways controlled by protein kinase A (Pka1).

In addition, the authors have provided an updated Fig 4 with the correct results and correct figure legend. Please see the complete, correct Fig 4 caption here:

**Fig 4:**
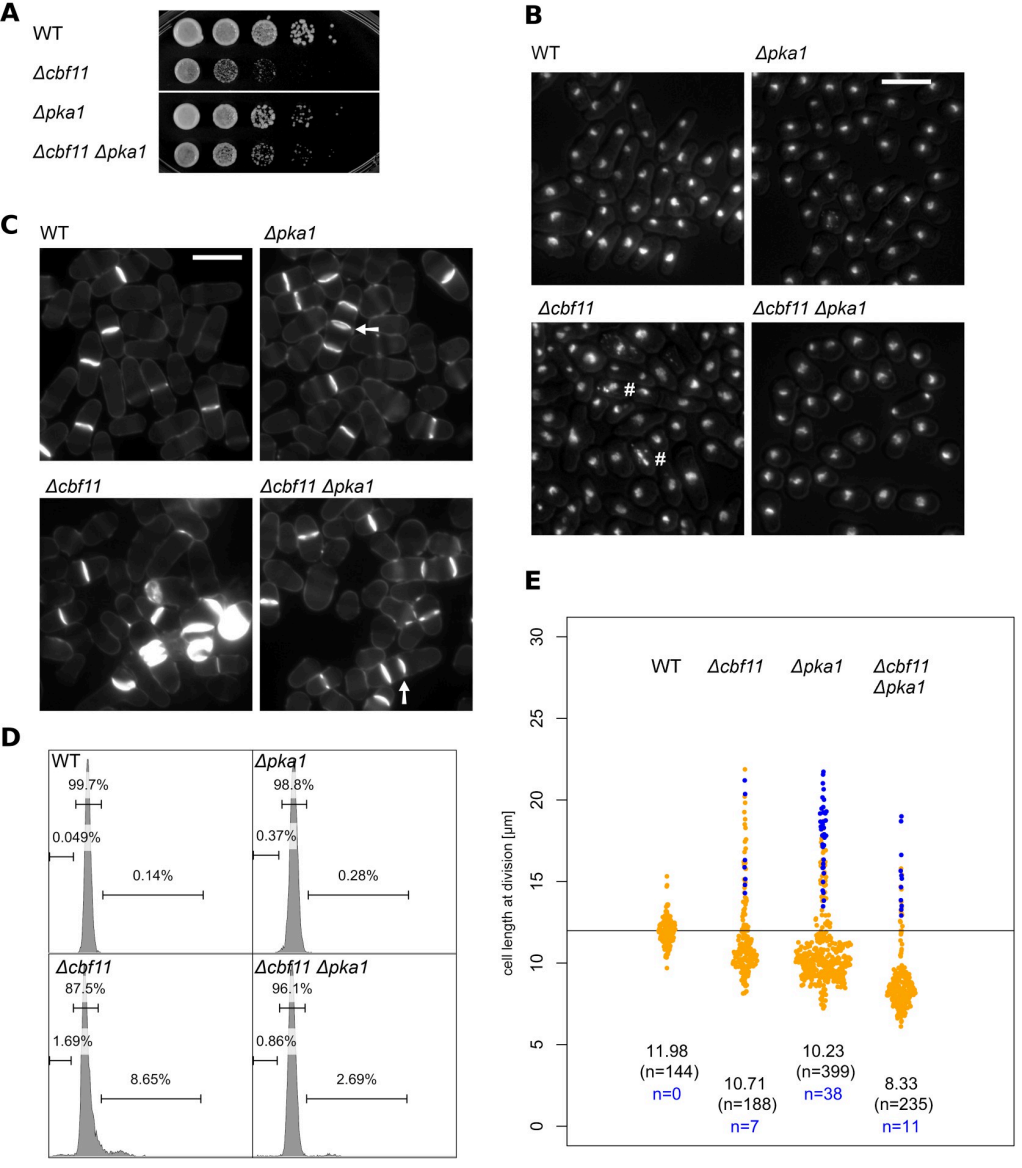
*Cbf11* interacts genetically with the Pka1 pathway. **(A)** 10-fold serial dilutions of cultures with the indicated genotypes were spotted on YES plates and grown for 2 days. The slow growth phenotype of *Δcbf11* cells is moderately suppressed by the deletion of *pka1*. **(B)** Cells growing exponentially in YES were fixed and stained with DAPI. Nuclear integrity defects of *Δcbf11* cells (marked with ‘#’) are diminished by deletion of *pka1*. The DAPI signal was overlaid with the corresponding DIC image to visualize cell contours. Scale bar 10 μm. **(C)** Calcofluor staining documents that the occurrence of *Δcbf11*-associated septation defects (e.g., single cells with multiple septa) is decreased in the double mutant with *Δpka1*. Multicellular filaments are marked by arrows. Scale bar 10 μm. **(D)** Flow cytometry analysis of DNA content in fixed, propidium iodide-stained cells grown to the exponential phase in YES. Deletion of *cbf11* results in aberrant DNA content distribution, which is, in part, corrected by deletion of *pka1*. Fractions of cells with <2C, 2C, and >2C DNA content are indicated in the histograms. **(E)** The length of fully septated cells from (C) was measured. Deletion of *pka1* has marked influence on the length of *Δcbf11* cells. Each dot represents a single cell measurement; median length and *n* values are indicated above each distribution; WT median value is indicated as a black horizontal line. Data points corresponding to short, multicellular filaments and their *n* values are shown in blue.
