# Supplementary figures and images for: Fission Yeast CSL Transcription Factors: Mapping Their Target Genes and Biological Roles
Source: PLoS One. 2015 Sep 14;10(9):e0137820. doi: 10.1371/journal.pone.0137820 (PMC4569565; doi:10.1371/journal.pone.0137820)

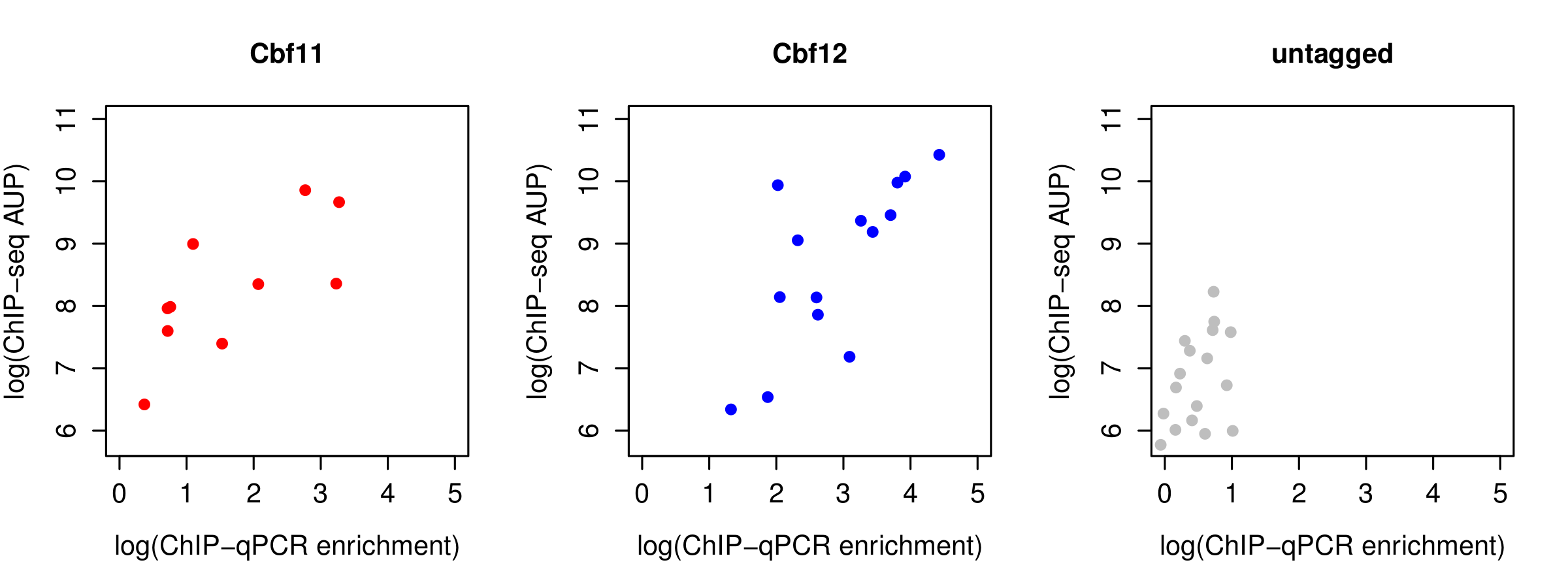

Supplement: S2 Fig — Selected CSL binding sites identified from ChIP-seq data by peak calling algorithm MACS [42] were validated by ChIP-qPCR. The ChIP-qPCR enrichment of target loci DNA was normalized to a control locus where no CSL binding was detected by ChIP-seq and plotted against integrated ChIP-seq signal from the region ±100 bp from qPCR amplicon centre (area under peak, AUP). (TIF) [file pone.0137820.s002.tif]

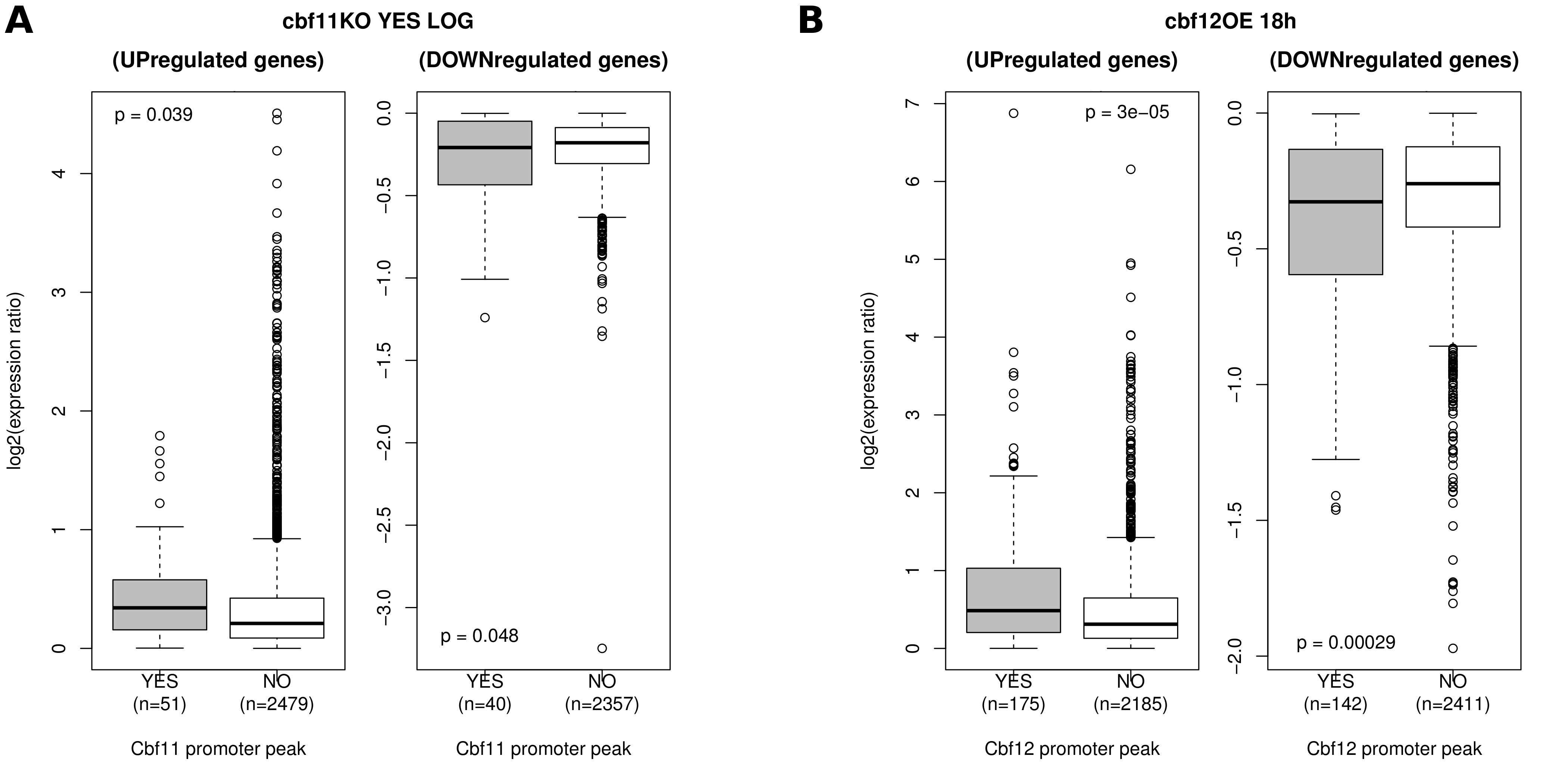

Supplement: S3 Fig — (A) Average expression values were calculated from all biological replicates of Δcbf11 cells growing exponentially in YES. Genes were then divided into upregulated (average expression ratio to wild type > 1) and downregulated (average expression ratio to wild type < 1), and further classified by the presence or absence of Cbf11 binding in their promoter. Cbf11-bound genes typically show more pronounced changes in expression compared to other genes. P-values of one-tailed t-test are indicated. (B) An analogous analysis as in (A) performed for Cbf12 target genes under cbf12 overexpression (18 hrs). Again, Cbf12-bound genes typically display more differential expression compared to all other genes. (TIF) [file pone.0137820.s003.tif]

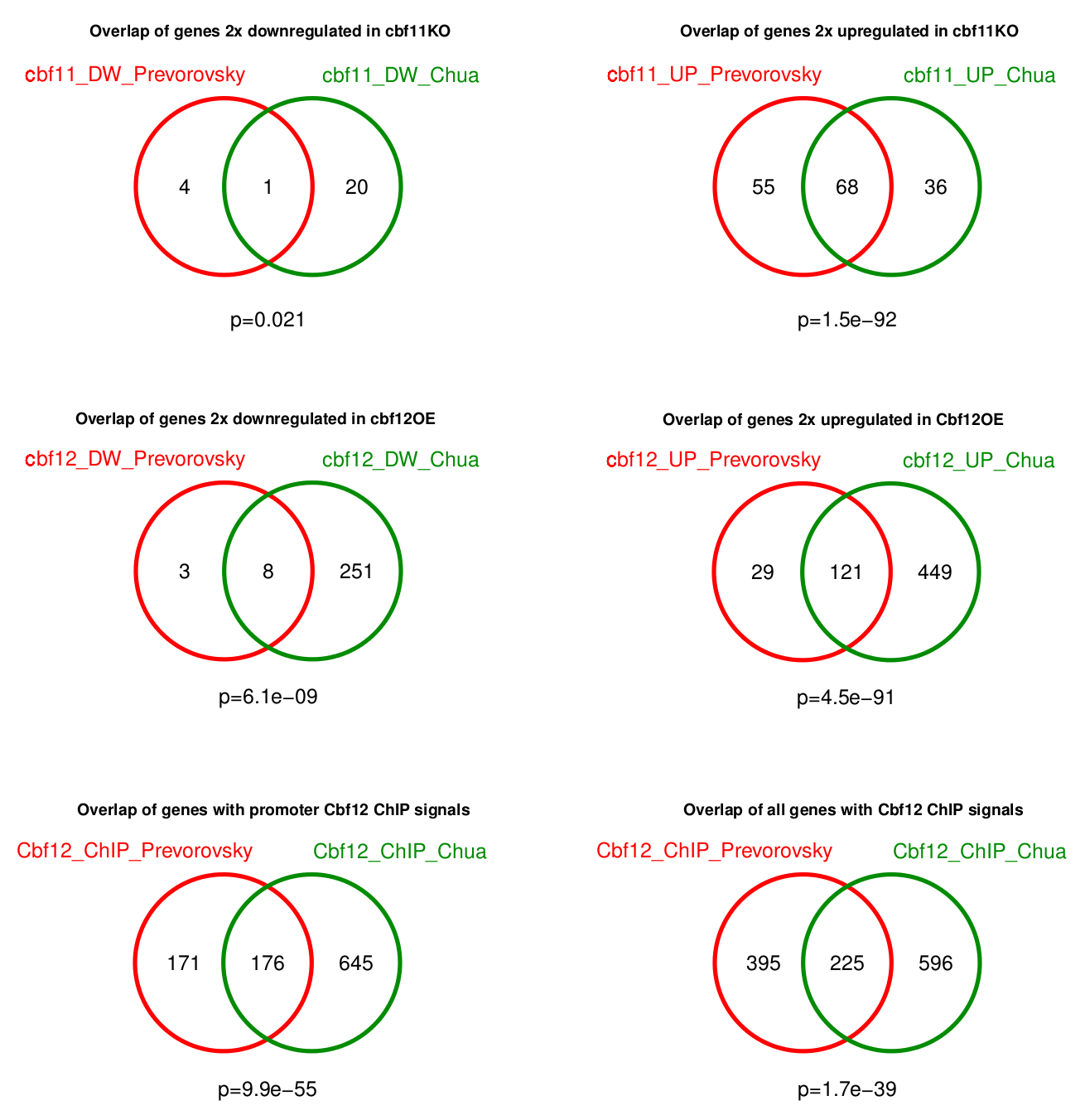

Supplement: S4 Fig — Comparison of CSL DEGs and CSL-bound genomic loci between this study (red) and Kwon et al. (green) [24]. Overlap significance was determined using the Fisher's exact test. (TIF) [file pone.0137820.s004.tif]

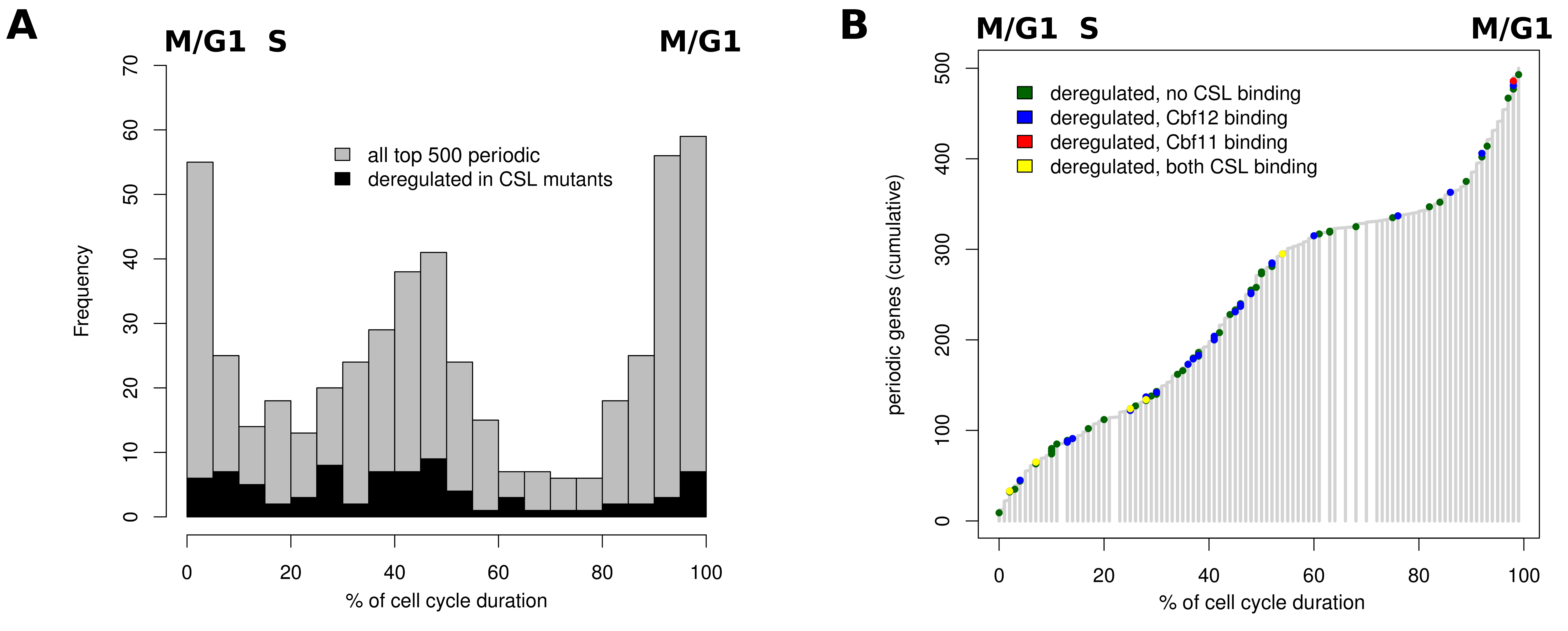

Supplement: S5 Fig — (A) Histogram of peak expression times for all top 500 periodic genes (grey) and for the subset thereof that is deregulated in CSL mutants (black). Normalized peak expression times are plotted as the percentage of cell-cycle duration [47]. Cell-cycle phases are indicated on top. (B) Cumulative distribution of peak expression times for all top 500 periodic genes (grey). Periodic genes showing deregulation in CSL mutants are highlighted as coloured dots. (TIF) [file pone.0137820.s005.tif]

**A**

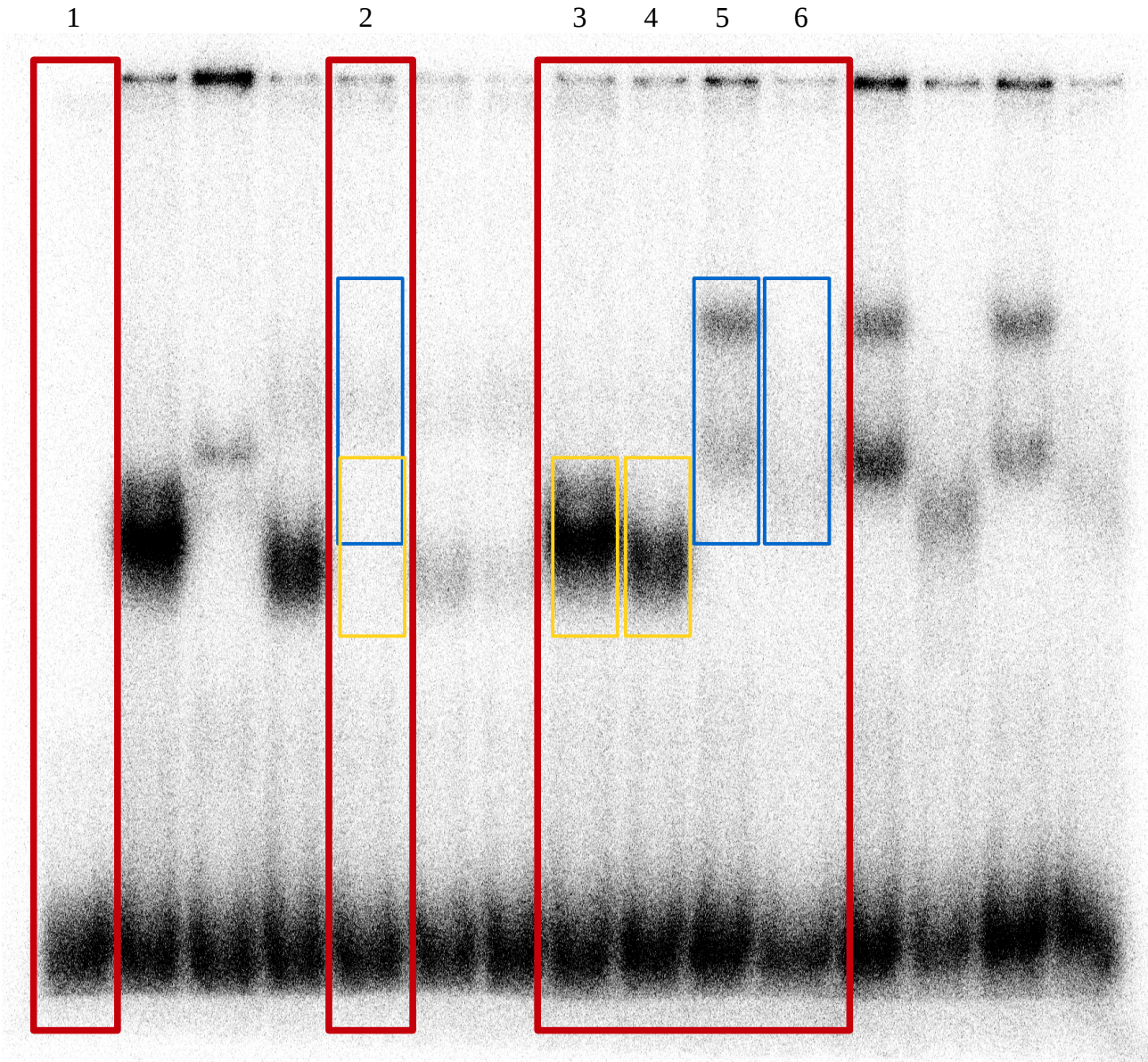

**B**

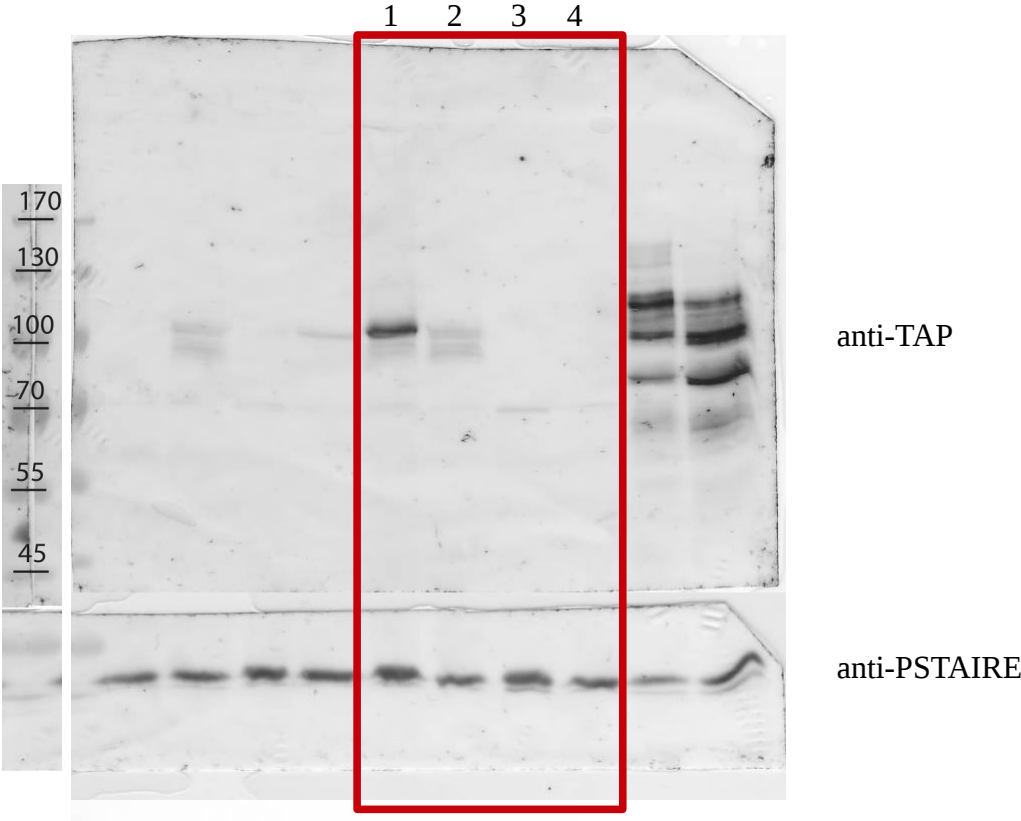

C

top panel

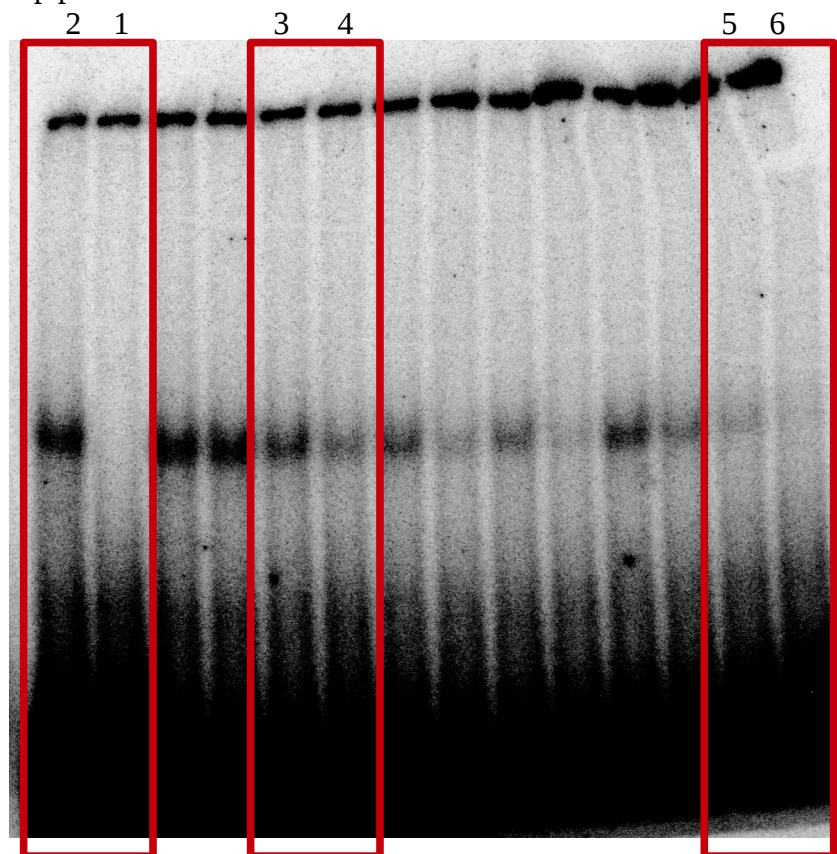

bottom panel

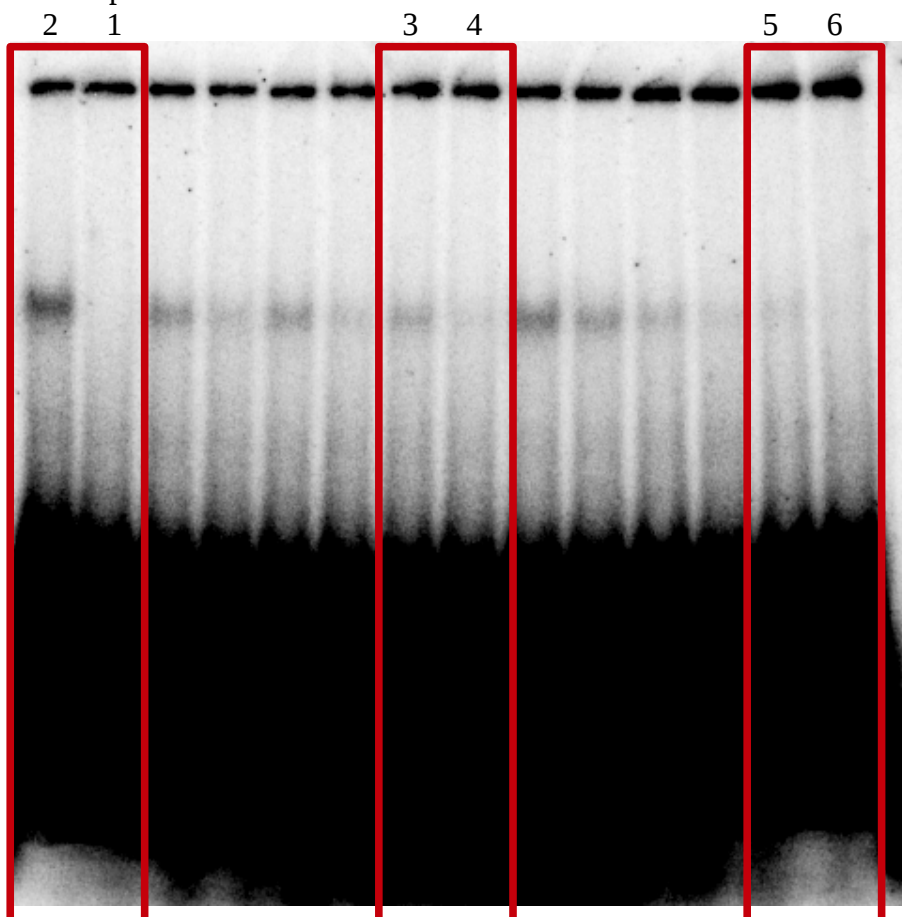

Supplement: S1 Gels — (A) EMSA from Fig 5F. Relevant lanes used for Fig 5F are highlighted in red. Blue and yellow rectangles correspond to regions used for densitometry. (B) Western blot used for Fig 5G. Relevant lanes used for Fig 5G are highlighted in red. (C) EMSAs from Fig 6E. Relevant lanes used for Fig 6E are highlighted in red. (PDF) [file pone.0137820.s006.pdf]
